# CaSR Antagonist (Calcilytic) NPS 2143 Hinders the Release of Neuroinflammatory IL-6, Soluble ICAM-1, RANTES, and MCP-2 from Aβ-Exposed Human Cortical Astrocytes

**DOI:** 10.3390/cells9061386

**Published:** 2020-06-02

**Authors:** Anna Chiarini, Ubaldo Armato, Peng Hu, Ilaria Dal Prà

**Affiliations:** 1 Human Histology and Embryology Section, Department of Surgery, Dentistry, Pediatrics and Gynecology, Medical School, University of Verona, Veneto, 37134 Verona, Italy; ubaldo.armato@univr.it (U.A.); peng.hu@univr.it (P.H.); 2 Burns Department, Shenzhen Second People’s Hospital, University of Shenzhen, Shenzhen 518000, China

**Keywords:** astrocytes, human, calcium-sensing receptor, IL-6, ICAM-1, RANTES, MCP-2, amyloid-β, neuroinflammation, neurodegeneration

## Abstract

Available evidence shows that human cortical neurons’ and astrocytes’ calcium-sensing receptors (CaSRs) bind Amyloid-beta (Aβ) oligomers triggering the overproduction/oversecretion of several Alzheimer’s disease (AD) neurotoxins—effects calcilytics suppress. We asked whether Aβ•CaSR signaling might also play a direct pro-neuroinflammatory role in AD. Cortical nontumorigenic adult human astrocytes (NAHAs) in vitro were untreated (controls) or treated with Aβ_25–35_ ± NPS 2143 (a calcilytic) and any proinflammatory agent in their protein lysates and growth media assayed via antibody arrays, enzyme-linked immunosorbent assays (ELISAs), and immunoblots. Results show Aβ•CaSR signaling upregulated the synthesis and release/shedding of proinflammatory interleukin (IL)-6, intercellular adhesion molecule-1 (ICAM-1) (holoprotein and soluble [s] fragment), Regulated upon Activation, normal T cell Expressed and presumably Secreted (RANTES), and monocyte chemotactic protein (MCP)-2. Adding NPS 2143 (i) totally suppressed IL-6′s oversecretion while remarkably reducing the other agents’ over-release; and (ii) more effectively than Aβ alone increased over controls the four agents’ distinctive intracellular accumulation. Conversely, NPS 2143 did not alter Aβ-induced surges in IL-1β, IL-3, IL-8, and IL-16 secretion, consequently revealing their Aβ•CaSR signaling-independence. Finally, Aβ_25–35_ ± NPS 2143 treatments left unchanged MCP-1′s and TIMP-2′s basal expression. Thus, NAHAs Aβ•CaSR signaling drove four proinflammatory agents’ over-release that NPS 2143 curtailed. Therefore, calcilytics would also abate NAHAs’ Aβ•CaSR signaling direct impact on AD’s neuroinflammation.

## 1. Introduction

Alzheimer’s disease (AD) is the world’s most prevalent form of dementia [1]. Global population aging has increased its incidence, making AD a serious familial, healthcare, and societal burden. The main AD’s neuropathology hallmarks are amyloid-β (Aβ) senile plaques, hyperphosphorylated Tau (hp-Tau) protein neurofibrillary tangles (NFTs), and a chronic diffuse neuroinflammation due to activated innate immune pathways in glial cells [2,3]. The inexorably spreading neuropathology causes a worsening neural circuitry breakdown due to the slowly escalating death of neurons and oligodendrocytes, which eventually causes the patients’ memory loss, cognitive decline, and ultimate demise [4,5]. Previously, the mainstream research focused on the pathogenetic roles played by Aβ peptides (Aβs) and hp-Tau proteins, the two main AD drivers [4,5]. More recently, AD’s neuroinflammation mechanisms have been attracting increasing attention [2,3]. The brain’s growing load of soluble Aβ oligomers drives the activation of astrocytes and microglia. Based on objective facts, some authors posit that the induced reactive astrogliosis plays a prominent role in AD’s neuroinflammation [6]. In fact, astrocytes are the most abundant brain cell type (from 1.7 to 2.2 fold and more the neurons’ number), and the timescale of astrocytes’ proinflammatory signaling lasts longer than that of the less abundant microglia [7,8]. However, Aβ-activated astrocytes and microglia reciprocally interact with each other by releasing a complex set of agents that sustain and spread the neuroinflammation [9]. Here, it is worth pointing out that human cerebral cortex astrocytes do differ under significant aspects from their rodent counterparts, e.g., cell subtypes, size, primary processes numbers, gap junctions-connected networks, tripartite synapses assembling and disassembling, modulation of neurons’ metabolism and functions, physiological blood-brain barrier (BBB) roles, and transcriptome profiles [10,11,12,13,14]. Moreover, human astrocytes more intensely perform intricate metabolic tasks, e.g., Ca^2+^ waves propagation, secretion and/or uptake of neurotransmitters, gliotransmitters, neuromodulators, hormones, metabolic, trophic, and plastic factors, than rodents’ astrocytes do (see for references, [10,15]). Due to a variety of reasons, astrocytes isolated from rodent models have hitherto been the preferred experimental models for AD studies. However, it cannot be disregarded that the evolutionary changes the human brain has undergone prevent AD-model animals from fully mirroring human AD. These hard facts underscore the persistently failed translation of promising anti-AD drugs from AD-model animals to human AD patients [6,16,17]. Human primary adult astrocytes are isolated directly from brain cortex and retain the morphological and functional characteristics of their tissue of origin, so they are reputed the cell culture model that more closely represents the human in vivo situation being useful to investigate basic biological processes, or manipulate cellular functions and behaviors. It can be argued that the experimental exploitation of preclinical “in Petri dishes” AD models involving cortical normal (untransformed) adult human astrocytes (NAHAs) and/or neurons and/or microglia is likely to yield results closer to the human brain’s physiological or pathological conditions [17].

The calcium-sensing receptor (CaSR), a member of Family C G-protein-coupled receptors (for more details and relevant references see [18,19]), quickly senses changes in extracellular Ca^2+^ levels [Ca^2+^]_e_, yet also binds other cations such as polyamines, amino glycoside antibiotics, and Aβs [15,20,21,22]. CaSR’s intracellular domains interact with various G-proteins to enact a complex of signaling activities, consisting of (i) protein kinases (AKT, PKCs, and MAPKs); (ii) phospholipases (A2, C, and D); (iii) transcription factors (TFs); (iv) second messenger production (e.g., cyclic AMP); and (v) Ca^2+^ influx via TRPC6-encoded receptor-operated channels [23,24]. All central nervous system (CNS) cell types express CaSRs, though expression levels differ by region, with distinctly high expression in the hippocampus [25]. As revealed by RT-PCR and western blot analysis, primary human embryo astrocytes express both CaSR mRNA and protein. Meningiomas and astrocytomas too express similar levels of CaSR as appraised by northern, RT-PCR, and western blot analyses [26]. Moreover, mitotically quiescent nontumorigenic (“normal”) adult human astrocytes (NAHAs) express functional CaSRs more intensely than proliferating astrocytes independent of [Ca^2+^]_e_ changes [27]. Apart from controlling general Ca^2+^ homeostasis (calciostat function) by modulating parathyroid hormone (PTH) release, renal Ca^2+^ reabsorption, and skeletal Ca^2+^ storage [24,28], CaSRs play extra relevant roles in the CNS, physiologically modulating K^+^ fluxes [29], L-amino acid sensing [30], neural cells growth, differentiation, prenatal migration, and postnatal synaptic neurotransmission [25,31,32,33,34]. Changes in expression or altered function of CaSRs also affects CNS diseases, such as AD and ischemia/hypoxia/stroke, by regulating outward K^+^ channel fluxes, nitric oxide (NO) and vascular endothelial growth factor A (VEGF-A) overproduction, amyloidogenesis, glial activation, and neuronal death [18,20,21,35,36,37,38].

The use of cortical NAHAs and postnatal HCN-1A neurons as in vitro preclinical models has brought to light a novel pathological interaction by which exogenous Aβs bind and activate CaSRs [20,21,35] being the Aβ•CaSR complexes subsequently endocytosed in NAHAs [15,35]. Apart from the transient overexpression of the CaSR in NAHAs and the progressive overexpression in the hippocampal astrocytes and neurons of 3 × Tg AD-model mice [20,39], this interaction drives the surplus production, accumulation, and secretion of Aβ_42_ and hyperphosphorylated Tau oligomers from both NAHAs and neurons, coupled with increased cell death among the latter. The CaSR agonist NPS R-568 increases Aβ_42_ oligomers release from NAHAs, mirroring the effect of Aβ_25–35_•CaSR signaling [20,21]. At the same time, Aβ•CaSR signaling in astrocytes brings about the surplus synthesis/release of NO and VEGF-A [27,35]. Such results implied that diverse phenylalkylamines, functioning as CaSR negative allosteric modulators (NAMs or calcilytics; e.g., NPS 89636 and NPS 2143) able to move the CaSR response curve to alterations of [Ca^2+^]_e_ [22,24] may be prospective AD medicaments. Calcilytic NPS 2143 stifled all previously mentioned neurotoxic effects driven by exogenous Aβ_25–35_•CaSR signaling and fully preserved neuronal viability [20,21,35].

Several reports have linked the CaSR to tissue inflammation and vice versa. In general, CaSR’s expression and calciostat function is upregulated by proinflammatory cytokines like interleukin (IL)-1β and IL-6 produced as part of the innate immune response to tissue damage and inflammation. On its own part, the CaSR advances the inflammatory response through multiprotein inflammasomes that trigger caspase-1 to produce IL-1β [40]. Lines of evidence indicate the CaSR’s involvement in the cytokine-mediated inflammation ensuing deep burn wounds [40], at the level of the airways of human allergic asthma patients and of allergen-sensitized mice [41], in adipose tissue [42], colon-rectum [43], prostate [44], lipopolysaccharide (LPS)-treated lungs [45], and kidneys after ischemia and reperfusion in streptozotocin-induced diabetic rats [46]. In fact, CaSR NAM NPS 2143 administration did elicit an effective anti-inflammatory action in asthma [41] and in LPS-evoked pneumonia [45].

As other laboratories reported, Aβ-exposed animal and human astrocytes release various proinflammatory cytokines [3,9,47,48,49,50] and chemokines [51,52,53]. Therefore, in this work we investigated whether Aβ•CaSR signaling might also partake in the astrocytes’ excess production and secretion of neuroinflammatory agents besides the above-mentioned neurotoxins. The present results prove for the first time that NAHAs’ Aβ•CaSR signaling also drives the over-release of neuroinflammatory IL-6, soluble intercellular adhesion molecule-1 (s-ICAM-1), Regulated upon Activation, Normal T Cell Expressed and Presumably Secreted (RANTES), and Monocyte chemotactic protein (MCP)-2. Our findings also show that administering a CaSR NAM [22,24] nearly totally or considerably hinders the oversecretion of such neuroinflammatory agents, demonstrating further anti-AD beneficial effects of calcilytics to be added to the several ones previously reported [15,20,21,35,36,54].

## 2. Materials and Methods

### 2.1. Bioethics

NAHAs were isolated from anonymized surgical fragments of normal adult human temporal cortex (brain trauma leftovers) provided by several Neurosurgery Units after obtaining written informed consent from all the patients and/or their next-of-kin. Experimental use of isolated NAHAs was approved by the Ethical Committee of Verona’s University-Hospital Integrated Company, Prog. No. CE118CESC. The ethical approval was obtained on the 1st April 2014 with Protocol number 15877. All human cells experiments were performed in accordance with the relevant guidelines and regulations of Verona’s University-Hospital Integrated Company.

### 2.2. Isolation and Culture of Phenotypically Locked-in Nontumorigenic Adult Human Astrocytes (NAHAs)

**Figure 1:**
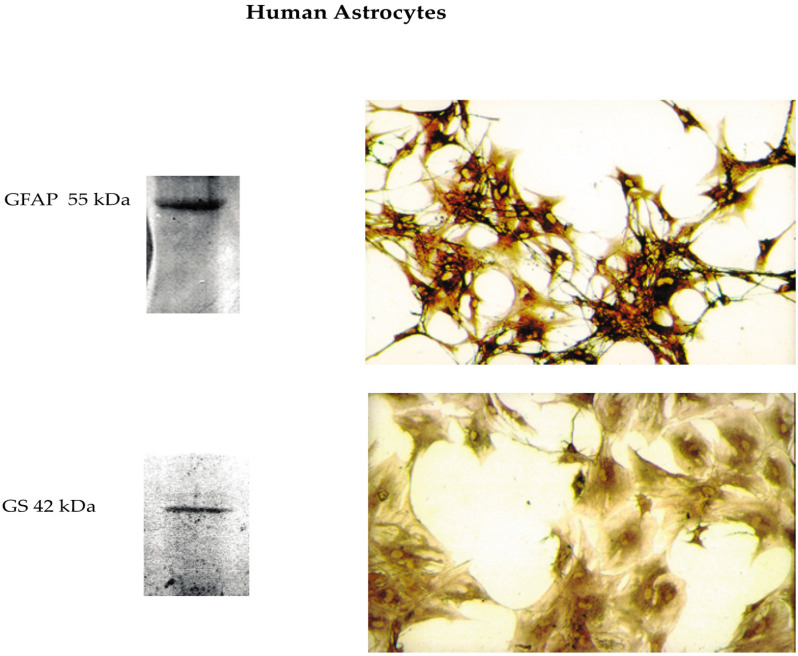
Pictures of pure (100%) in vitro cultures of cortical nontumorigenic adult human astrocytes (NAHAs) that express their cell type-specific markers glial fibrillary acid protein (GFAP) (top panels) and glutamine synthase (GS) (bottom panels) as detected in immunoblots (left panels) and via immunocytochemistry (right panels). NAHAs cultures were set up as detailed in the Materials and Methods). After two weeks of staying in vitro, they were sampled and processed for western immunoblotting and immunocytochemistry as described in the Materials and Methods. Original magnification of the microscope pictures: GFAP, X 100; GS, X 200.

Untransformed NAHAs were isolated as previously detailed [21] from temporal lobe cerebral cortex leftovers of five male patients (range: 18–38 years) with perforating head injuries due to motorcycle accidents who underwent hasty neurosurgery. Briefly, the leftovers were dipped into MCDB 153 medium (Sigma-Aldrich, Milan, Italy), put into a Dewar flask at 4 °C, and carried to the laboratory. There they were soon cut into tiny pieces. The cells were released via a mild treatment with 0.25% (*w/v*) trypsin (Gibco, Thermo Fisher Scientific, Monza, MB, Italy) in Hank’s Basal Salt Solution (BSS; Gibco, Thermo Fisher Scientific) at 18 °C and the residual pieces were triturated with a series of Pasteur pipettes with decreasing (from 5 to 1 mm) bore diameters. The isolated cells were planted in 25 cm^2^ culture flasks (BD Biosciences, Le Pont de Claix, France) containing 2 mL of a medium consisting of 89% (*v/v*) of a 1:1 mixture of Ham’s F-12 and MCDB 153 media (Sigma-Aldrich,), 10% (*v/v*) heat-inactivated (at 56 °C for 30 min) fetal bovine serum (FBS; Gibco, Thermo Fisher Scientific), and 1% (*v/v*) of a penicillin–streptomycin solution (Gibco, Thermo Fisher). Basic fibroblast growth factor (bFGF or FGF-2; 20 ng mL^−1^; PeproTech EC Ltd., London, UK), insulin-like growth factor-I (IGF-I; 20 ng mL^−1^; PeproTech), platelet-derived growth factor (PDGF; 20 ng mL^−1^; PeproTech), and epidermal growth factor (EGF; 10 nM; Sigma-Aldrich) were added to the medium to enhance the initial proliferation and selection of the astrocytes in the mixed cell population. This complete medium was replaced every 2–3 days. When the primary mixed cultures became 70% confluent (after 1–4 weeks), the cells were detached from the flask surfaces with 0.25% (*w/v*) trypsin and 0.02% (*w/v*) EDTA (Gibco, Thermo Fisher) in Hank’s BSS, split 1:4 and planted in new flasks. After the third subculture, a pure (100%) population of astrocytes was obtained that no longer needed growth factors. Immunocytochemistry and western immunoblot analysis of the cells of these pure cultures revealed the expression of only astrocyte-specific markers, such as glial fibrillary acid protein (GFAP) and glutamine synthase (GS) (Figure 1). No cells expressed neurons’ (enolase), microglia’s (CD-68), oligodendrocytes’ (galactocerebroside), or endothelial cells’ (factor VIII) markers. The astrocytes kept proliferating slowly (doubling time, 2–3 weeks) and expressing their characteristic markers in 90% (*v/v*) Ham’s F12/MCDB 153 medium (Gibco, Thermo Fisher) and 10% (*v/v*) heat-inactivated fetal bovine serum (Gibco, Thermo Fisher) with no growth factors added. They stopped growing but kept expressing their distinctive markers upon reaching confluence or after they were incubated in high-Ca^2+^ (1.8 mM) Dulbecco’s Modified Eagle Medium (DMEM, Gibco, Thermo Fisher). Thus, they were phenotypically “locked-in”. The proliferatively quiescent cells in confluent astrocyte cultures started cycling again when subcultured. The astrocytes kept expressing the CaSR both when they proliferated and 1.6-fold more intensely (*p* < 0.002) when they became mitotically quiescent after the exposure to the 1.8 mM Ca^2+^-containing DMEM. On the other hand, astrocytes’ CaSR expression levels were independent of the actual levels of extracellular Ca^2+^ [27]. Moreover, astrocytes’ CaSRs specifically bound exogenous Aβs, and the Aβ•CaSR complexes thus formed were quickly internalized [15,35,55]. At least 15–18 subcultures could be obtained over 2.5 years from a tiny piece (3–4 mm^3^) of normal cortex. Only astrocytes from the fourth to the eighth subculture were used for the experimental work.

### 2.3. Aβ Peptides

Aβ_25–35_ (Bachem AG, Bubendorf, Switzerland), a known Aβ_1–42_ proxy [56], was dissolved at 1.5 mM in PBS. Fibrillogenesis by Aβ_25–35_ was checked via thioflavin-T tests before experimental use. The reversemer peptide Aβ_25–35_ (Bachem) was dissolved in the same way as Aβ_25–35_, yet it did not form fibrils and when given to the NAHAs cultures was ineffective (not shown).

### 2.4. Experimental Protocol

Because astrocytes do not normally proliferate in the adult human brain, as in earlier works [15,20,21,35,36,52], we used confluent, proliferatively quiescent, NAHAs pure cultures in 1.8 mM Ca^2+^ DMEM (Gibco, Thermo Fisher). At experimental “0 h”, culture flasks (10^6^ NAHAs each) served partly as untreated controls receiving a change of fresh medium and partly received fresh medium with 20 µM of fibrillar (f)Aβ_25–35_. Exposure of NAHAs to fAβ_25–35_ lasted for the entire duration of experiments. This dose of fAβ_25–35_ had been found to be ideal in earlier studies [19,20]. The CaSR allosteric antagonist (calcilytic) NPS 2143 HCl (2-chloro-6-[(2 R)–3-1,1-dimethyl-2-(2-naphtyl)-ethylamino-2-hydroxy-propoxy]-benzonitrile HCl; Tocris Bioscience, Bristol, UK) was dissolved in DMSO and next diluted in the growth medium at a final concentration of 100 nM. At experimental “0 h”, “24 h”, “48 h”, and “72 h”, part of the fAβ_25–35_ astrocyte cultures was exposed for 30 min to NPS 2143 dissolved in fresh medium. Next, the NPS 2143-containing medium was removed and fresh (at 0.5 h) medium or the previously astrocyte-conditioned (at 24.5 h, 48.5 h, and 72.5 h) media were added again to the cultures. Cultured NAHAs and growth media were sampled at 24 h, 48 h, 72 h, and 96 h after the onset of each treatment. Phosphoramidon (10 μM; Sigma), an inhibitor of thermolysin and other proteases, was added to the media at “0 h” experimental time.

### 2.5. Immunocytochemistry

Immunostaining of astrocytes, which had been seeded into 24-well plates for primary tissue cultures (Becton-Dickinson, Franklin Lakes, NY, USA), was carried out at 4 °C. Astrocytes (2.0 × 10^4^/chamber) were washed twice with PBS (phosphate-buffered saline) containing BSA (1.0% *w/v*) and NaN_3_ (0.1% *w/v*), and incubated for 60 min at room temperature with mouse monoclonal antibodies (at 1.0 μg mL^−1^) against GFAP and GS (both from Santa Cruz Biotechnology Inc., Heidelberg, Germany). The cells were washed three times with PBS-BSA solution, next incubated for 60 min at room temperature with specific secondary antibodies conjugated to horseradish peroxidase (all from Santa Cruz Biotechnology). Specific immunostainings were developed with 3, 3′-diaminobenzidine (Sigma-Aldrich). After a final wash with PBS-BSA solution, specimens were examined under an inverted Zeiss IM35 microscope (Carl Zeiss Vision Italia, Milan, Italy) and photographed with an Olympus 3300^TM^ (Olympus Life Sciences, Milan, Italy) digital camera. Appropriate parallel controls were run with no primary or secondary antibody.

### 2.6. Antibody Array

We found and quantified the proinflammatory cytokines and chemokines detectable in NAHAs-conditioned media (Table 1) by using the Cytokine Kit RayBio^TM^ Array 3 (RayBiotech, Inc., Peachtree Corners, GA, USA), according to the manufacturer’s protocols. Briefly, NAHAs were treated for 48 h and 96 h with fAβ_25–35_ 20 µM ± NPS 2143 100 nM. NPS 2143 is a well-established highly selective and specific NAM of the CaSR [22,24]. A total of 2 mL of NAHAs-conditioned media sampled 48 h or 96 h after the onset of the treatments was incubated with the antibody array membranes previously treated for 30 min with Odissey^TM^ blocking buffer (LI-COR Biosciences, Lincoln, NE, USA). Next, after a 2 h incubation at room temperature, the array membranes were thoroughly washed and incubated for 2 h with 1.0 mL of primary biotin-conjugated antibody, diluted 1:250 in Odissey^TM^ blocking buffer. Finally, the membranes were incubated at room temperature for 1 h with 2 mL of DyLight800-conjugated streptavidin (KPL; SeraCare Life Sciences, Milford, MA, USA) diluted 1:7500 in Odissey^TM^ blocking buffer. The positive signals of the detected cytokines and chemokines were acquired with an Odissey^TM^ (LI-COR Biosciences) scanner and later quantified using the Image Studio^TM^ (version 5.2; LI-COR Biosciences) software. The intensities of the positive signals from each array were normalized via comparisons to corresponding positive controls.

### 2.7. Enzyme-Linked Immunosorbent Assays (ELISAs) of IL-6, MCP-2, RANTES, and Soluble ICAM-1 Released into NAHAs-Conditioned Growth Media

To begin, 1 × 10^6^ NAHAs were seeded in 25 cm^2^ flasks and cultured with 4 mL of medium, (two flasks for each experimental time point). We sampled NAHAs-conditioned growth media taken at 0 h, 24 h, 48 h, 72 h, and 96 h of exposure to fAβ_25–35_ 20 μM ± NPS2143 and directly stored them at −80 °C to be later assayed for their IL-6 or MCP-2 or RANTES or soluble (s)-ICAM-1 contents. The same cell-conditioned medium samples were tested for assessing IL-6, RANTES, MCP-2 and s-ICAM-1 by means of different ELISA kit. Five independent experiments were repeated using NAHAs from as many individuals. To do this we used the following commercial kits: Human IL-6 PicoKine^TM^ ELISA (Boster Biological Technology Co., Ltd., Pleasanton, CA USA); RayBio^TM^ Human MCP-2 ELISA (RayBiotech); RayBio^TM^ Human RANTES ELISA (RayBiotech); and ICAM-1 (CD54) Human Simple Step ELISA (Abcam, Cambridge, UK). We carried out the tests according to the instructions of the respective manufacturers. The sensitivity of the assays was < 0.3 pg mL^−1^ for IL-6, 1.5 pg mL^−1^ for MCP-2, 3 pg mL^−1^ for RANTES, and 1.6 pg mL^−1^ for s-ICAM-1.

### 2.8. Western Immunoblotting

First, 1 × 10^6^ NAHAs were seeded in 25 cm^2^ flasks and cultured with 4 mL of medium, (two flasks for each experimental time point). At selected timepoints, we scraped untreated and treated NAHAs into cold PBS, sedimented them at 200*g* for 10 min, and homogenized the pellets in T-PER^TM^ tissue protein extraction reagent (Pierce, Rockford, IL) that included a complete EDTA-free protease inhibitor cocktail (Roche, Milan, Italy). We determined the protein contents of the samples by using the Bio-Rad Protein Assay (Bio-Rad). Briefly, equal amounts (20–30 µg) of protein from the lysates were heat-denatured for 10 min at 70 °C in a proper volume of 1× NuPAGE LDS sample buffer supplemented with 1× NuPAGE reducing agent (Invitrogen). Next, the lysates were loaded on a NuPAGE Novex 4–12% Bis-Tris polyacrylamide gel (Invitrogen, Life Technologies, Monza, MB, Italy). After electrophoresis in NuPAGE MES SDS running buffer using the Xcell SureLock^TM^ Mini-Cell (Invitrogen) (50 min run-time at 200 V constant), proteins were blotted onto nitrocellulose membranes using the iBlot^TM^ Dry Blotting System (Invitrogen). Membranes were probed with rabbit antihuman IL-6, or rabbit antihuman ICAM-1, or rabbit antihuman RANTES IgG polyclonal antibodies (all at a final dilution of 0.5 µg mL^−1^; Boster Biological Technology), or mouse antihuman MCP-2 antibody (at a final dilution 1:500; GeneTex Inc., Irvine, CA, USA), or mouse antihuman GFAP antibody and mouse antihuman GS antibody, or goat anti-lamin B1 antibody (at a final dilution of 1.0 µg mL^−1^; all from Santa Cruz Biotechnology Inc.). Subsequent processing steps were as previously detailed [20]. We used lamin B1 as the loading control. We carried out the densitometric analysis of the immunoblots’ specific protein using Image Studio^TM^ (version 5.2, LI-COR) software.

### 2.9. Statistical Analysis

Statistical analysis of the data was performed using SigmaStat^®^ 3.5 Advisory Statistics for Scientists (Systat Software, Richmond, CA) and Analyse-it (Analyse-it Software Ltd., UK). Densitometric data were normalized to lamin B1 (loading control) and next analyzed by one-way ANOVA. When ANOVA’s upshots were significant (*p* < 0.05) we used post hoc Tukey’s test for all pairwise comparisons and multiple comparisons vs. untreated control values. Null hypotheses were rejected when *p* < 0.05.

## 3. Results

### 3.1. Antibody Array-Detected Changes in Neuroinflammatory Agents Released from Untreated and fAβ_25–35_ ± NPS 2143-Treated NAHAs

**Figure 2:**
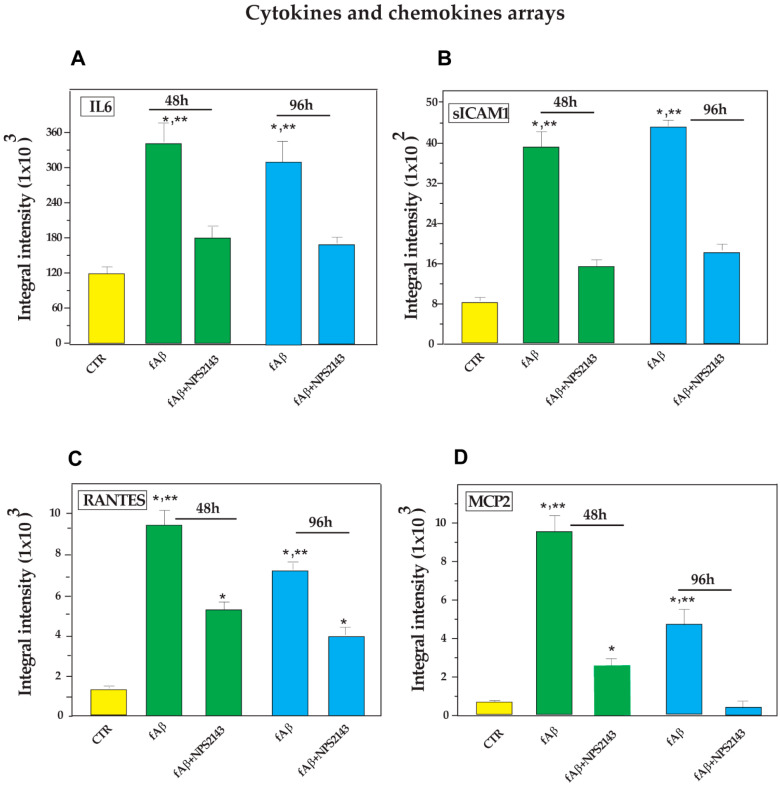
Time-dependent differential expression of (**A**) Interleukin (IL)-6, (**B**) Soluble intercellular adhesion molecule-1 (s-ICAM-1), (**C**) Regulated upon Activation, normal T cell Expressed and presumably Secreted (RANTES), and (**D**) Monocyte chemoattractant protein (MCP)-2, the basal secretion of which increased after treating NAHAs with fAβ_25–35_, yet significantly decreased after fAβ_25–35_ + NPS 2143 treatment. Each neuroinflammatory agent was detected in the NAHA-conditioned media via membrane-based antibody arrays as described in the Materials and Methods. The developed antibody arrays were analyzed in an Odissey^TM^ (LI-COR) scanner and the positive staining intensities of IL-6, s-ICAM-1, RANTES, and MCP-2 were quantified using the Image Studio^TM^ (version 5.2) software. The integral intensities of the positive signals from each array were normalized via comparisons to corresponding controls. Bars are means ± SEMs of three independent experiments, each carried out in duplicate. One-way ANOVA followed by post hoc Tukey’s test allowed to calculate *p* values. CTR, untreated controls. * *p* < 0.05 vs. CTR; ** *p* < 0.05 of fAβ_25–35_ alone vs. fAβ_25–35_ + NPS 2143 treatment.

The antibody array we used allowed us to assay 24 cytokines, 3 cytokine agonists, 12 chemokines, and 3 other agents, all related to inflammatory processes (see Materials and Methods and Table 1 for more details). Analysis of media conditioned by untreated (control) NAHAs sampled at both 48 h and 96 h after the onset of experiments detected the secretion of 11 compounds, that is four cytokines (i.e., IL-1β, IL-3, IL-6, and IL-16) [47,57,58], four chemokines (i.e., IL-8, MCP-1, MCP-2, and RANTES) [53,59,60,61,62], and three other compounds [i.e., metalloproteinase inhibitor-2 (TIMP-2), s-ICAM-1, and platelet-derived growth factor subunit-B (PDGF-B)] [63,64,65] (Table 1, Figure 2, and Figures S1 and S2 in Supplementary Materials).

The quantitative analysis of the antibody array results revealed that in the untreated NAHA-conditioned media, IL-6, MCP-1, IL-8, and TIMP-2 had the highest basal levels, whereas IL-1β, IL-3, IL-16, and MCP-2 had the lowest ones. Finally, RANTES and s-ICAM-1 had intermediate values (Figure 2 and v. Figure S1).

Treating NAHAs with fAβ_25–35_ alone significantly (*p* < 0.05 vs. controls) increased at 48 h and 96 h the release of cytokines IL-1β, IL-3, and IL-6, of chemokines IL-8, RANTES, and MCP-2, and also of s-ICAM-1 into the conditioned media (Figure 2 and v. Figure S1). Moreover, the exposure to fAβ_25–35_ elicited only at 48-h a significant surge (*p* < 0.05 vs. controls) of cytokine IL-16 (v. Figure S1).

Conversely, both at 48 h and 96-h, fAβ_25–35_ alone treatment did not change (*p* > 0.05 vs. controls) the basal secreted amounts of MCP-1, and TIMP-2 (v. Figure S1).

In summary, the antibody array results allowed us to distinguish three secretion patterns of the neuroinflammatory agents into the media from fAβ_25–35_ ± NPS 2143-treated NAHAs:

(i) a significant increase elicited by fAβ_25–35_ treatment, which fAβ_25–35_ + NPS 2143 cotreatment brought back to control levels by 96 h as was typical of IL-6, MCP-2, and soluble (s)-ICAM-1 fragment, whereas it only partially reduced RANTES secreted levels (Figure 2);
(ii) a significant increase brought about by fAβ_25–35_ exposure that did not change when adding NPS 2143 to fAβ_25–35_, as was proper of IL-1β, IL-3, and IL-8 at both 48 h and 96 h, whereas the surge of IL-16 at 48-h was only transient (v. Figure S1); and
(iii) no change vs. control levels after the addition of either fAβ_25–35_ by itself or fAβ_25–35_ + NPS 2143, as exemplified by MCP-1 and TIMP-2 (v. Figure S1).

These findings revealed for the first time that the AD-typical chronic neuroinflammation can be advanced by the NAHAs’ Aβ•CaSR signaling-elicited secretion of proinflammatory cytokine IL-6, chemokines RANTES and MCP-2, and of the s-ICAM-1 fragment.

### 3.2. Calcilytic NPS 2143 Effectively Hinders the Secretion of IL-6, MCP-2, RANTES, and s-ICAM-1 from NAHAs

To expand and validate the above antibody array findings, we used more sensitive ELISA tests to assay the amounts of IL-6, MCP-2, RANTES, and s-ICAM-1 fragment released into the growth media from NAHAs treated for 24 h, 48 h, 72 h, and 96 h with fAβ_25–35_ ± NPS 2143.

#### 3.2.1. IL-6 Secretion into NAHA-Conditioned Growth Media

**Figure 3:**
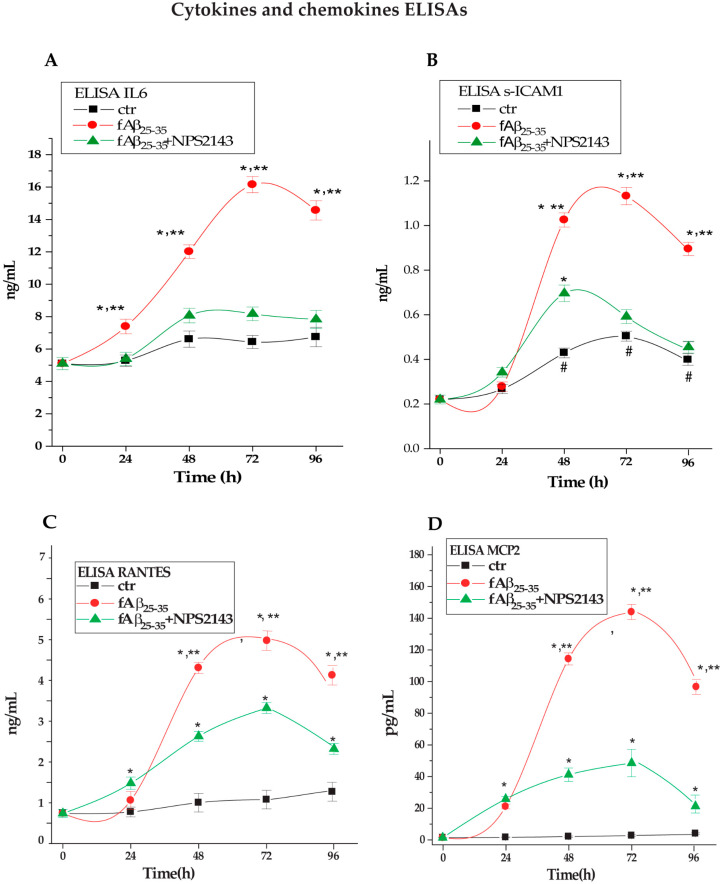
Changes in released levels of (**A**) IL-6, (**B**) s-ICAM-1, (**C**) RANTES, and (**D**) MCP-2 in NAHAs treated with fAβ_25–35_ ± NPS 2143 vs. untreated controls (CTR). Each agent was assessed in the NAHA-conditioned media via a specific ELISA kit as detailed in the Materials and Methods. Points on the curves are means ± SEMs of 3–5 independent experiments, each carried out in duplicate. One-way *ANOVA* followed by post hoc Tukey’s test allowed us to calculate *p* values. * *p* < 0.05 vs. time-corresponding CTR values; ^#^ *p* < 0.05 vs. 0-h CTR value; ** *p* < 0.05 vs. fAβ_25–35_ + NPS 2143 values.

The results of time-course ELISA assay revealed that Aβ•CaSR signaling directly and linearly increased the secreted amounts of IL-6 for up to 72 h, when it peaked at a 2.6-fold (*p* < 0.05) value over untreated controls and remained so high even at 96 h (Figure 3A). However, adding CaSR NAM NPS 2143 significantly (*p* < 0.05) suppressed at all time points the fAβ_25–35_-elicited IL-6 surplus secretion over parallel control values (Figure 3A). Accordingly, the cumulative (as estimated from the areas under the corresponding curves) 0 h to 96 h surplus release of IL-6 over control levels (+80.4%; *p* < 0.05) elicited by fAβ_25–53_ itself decreased to insignificance (+14.9%, *p* > 0.05 vs. controls; −81.5%, *p* < 0.05 vs. fAβ alone) when NAHAs were cotreated with fAβ_25–53_+NPS 2143 (Figure 3A; Table 2), thereby confirming the corresponding array data (cf. Figure 2). Therefore, pathological Aβ•CaSR signaling elicited most of the IL-6 oversecretion from NAHAs as calcilytic NPS 2143 effectively suppressed it.

#### 3.2.2. s-ICAM-1 Shedding into NAHA-Conditioned Growth Media

The time-course ELISA assay results showed that the amount of s-ICAM-1 that untreated NAHAs shed at 48 h, 72 h, and 96 h into the medium increased by 2.0/2.4-fold (*p* < 0.05) over 0-h and 24 h values (Figure 3B). On the other hand, during the first 24-h s-ICAM-1 shedding from fAβ_25–35_ alone-treated NAHAs did not significantly (*p* > 0.05) change vs. untreated control values. Subsequently, the fAβ_25–35_ treatment briskly raised s-ICAM-1 release making it reach by 48 h and 72 h 2.5-/2.4-fold higher values than parallel controls’ (*p* < 0.05); thereafter, it only slightly declined (96 h, 2.2-fold controls values, *p* < 0.05) (Figure 3B). Adding NPS 2143 to fAβ_25–35_ treatment significantly (*p* < 0.05 at all time points) curbed the Aβ•CaSR signaling-elicited s-ICAM-1 surplus shedding over basal (control) levels (Figure 3B). The cumulative (0 h to 96 h areas under the curves) s-ICAM-1 amount shed over control levels (+95.1%, *p* < 0.05) from the fAβ_25–__35_ alone-treated NAHAs was cut remarkably down by NPS 2143 addition (vs. controls, +34.0%, *p* < 0.05; vs. fAβ_25–__35_ alone, −62.3%, *p* < 0.05) (Table 2). Therefore, pathological Aβ•CaSR signaling was specifically responsible for the quota (62.3%) of the IL-6 surplus shed over control levels that calcilytic NPS 2143 suppressed.

#### 3.2.3. RANTES Secretion into NAHA-Conditioned Growth Media

As the time-course ELISA assay results showed, once exposed to fAβ_25–35_ alone, NAHAs secreted the same amounts of RANTES as controls did during the first 24 h. Next, fAβ_25–35_ alone-exposed NAHAs hugely increased RANTES release between 24 h and 72 h to moderately decrease it between 72 h and 96 h (48 h, +389%; 72 h, +355%; 96 h, +250%; *p* < 0.05 at each time point vs. untreated controls). Adding NPS 2143 partially yet significantly (*p* < 0.05 at each time point) curtailed the fAβ_25–__35_-elicited RANTES surplus secretion over controls’ baseline (Figure 3C). Thus, the cumulative (0 h to 96 h) fAβ_25–35_ alone-elicited RANTES surplus secretion over controls (+426.8%, *p* < 0.05) was considerably reduced (vs. controls, +248%, *p* < 0.05; vs. fAβ_25–__35_ alone, −41.9%, *p* < 0.05) in the media from fAβ_25–3_+NPS 2143-treated NAHAs (Table 2). Just as for s-ICAM-1, pathological Aβ•CaSR signaling specifically drove a conspicuous part (64.3%) of the RANTES surplus secretion from NAHAs that calcilytic NPS 2143 suppressed.

#### 3.2.4. MCP-2 Secretion into NAHA-Conditioned Growth Media

The time-course ELISA results showed that untreated (control) NAHAs steadily secreted very low basal MCP-2 amounts (1.9 pg mL^−1^) into the culture media. However, in keeping with corresponding protein array results, the treatment with fAβ_25–35_ alone hugely increased the secreted amounts of MCP-2 vs. untreated parallel controls (24 h, +1134%; 48 h, +6400%; 72 h, +8122%; 96 h, +5443%; *p* < 0.0001 at all time points). Conversely, after no change during the first 24 h, the NPS 2143 + fAβ_25–35_ cotreatment significantly cut down MCP-2 releases vs. fAβ_25–__35_ alone-treated values (48 h, +2186%; 72 h, +2357%; 96 h, +1043%; *p* < 0.0001 at all time points) (Figure 3D). Thus, the cumulative (0 h to 96 h) fAβ_25–__35_-elicited MCP-2 secretion surplus over controls (+3611.0%, *p* < 0.0001) significantly decreased with the NPS 2143 +fAβ_25–35_ treatment (vs. controls, +1361.9%, *p* < 0.05; vs. fAβ_25–__35_ alone, −62.3.9%, *p* < 0.05) (Table 2). Therefore, just as for s-ICAM-1 and RANTES, pathological Aβ•CaSR signaling specifically drove an important quota (62.3%) of the MCP-2 surplus secretion from NAHAs that NPS 2143 suppressed.

In conclusion, the Aβ•CaSR signaling involvement was shown by the ability of CaSR NAM NPS 2143 to significantly reduce the amounts of the just mentioned neuroinflammatory agents that NAHAs secreted/shed.

### 3.3. Changes in Cytokine/Chemokine Levels in NAHAs Protein Lysates Evoked by with fAβ_25–35_ ± NPS 2143 Treatments

#### 3.3.1. IL-6 Expression in NAHA Protein Lysates

**Figure 4:**
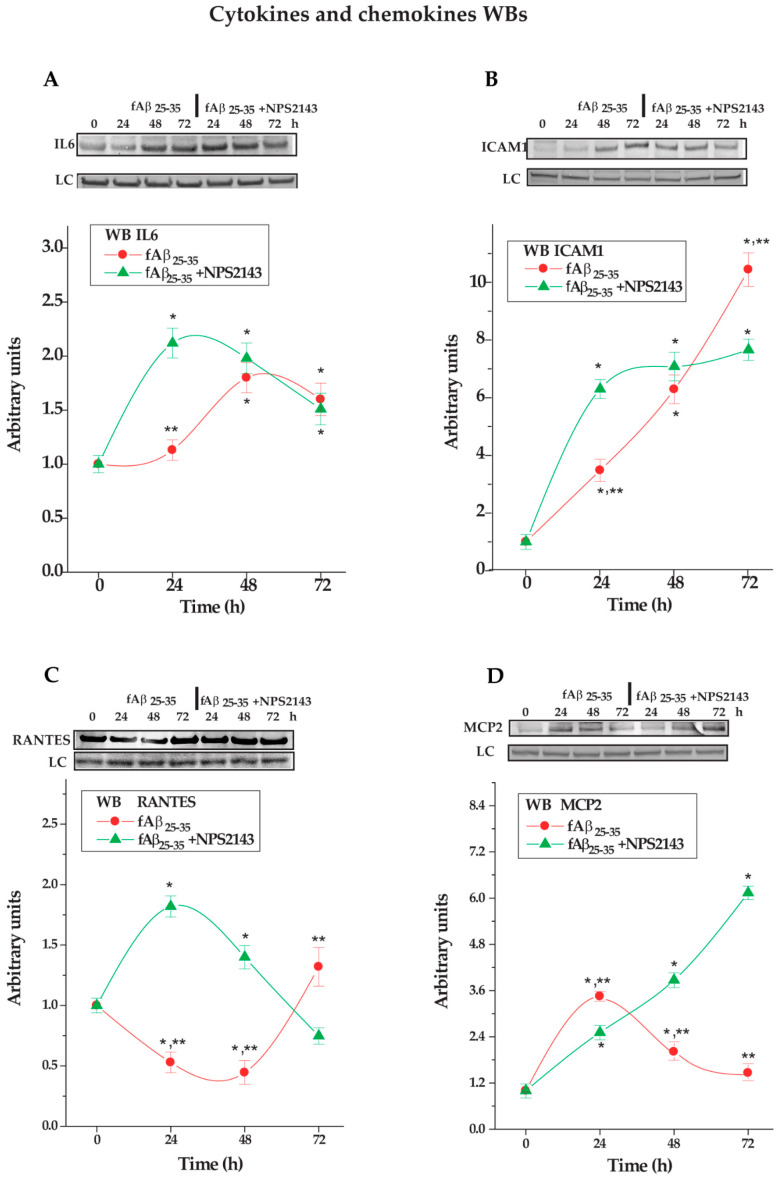
Changes in protein lysates’ levels of (**A**) IL-6, (**B**) s-ICAM-1, (**C**) RANTES, and (**D**) MCP-2 in NAHAs treated with fAβ_25–35_ ± NPS 2143 vs. untreated controls (CTR). At the top of each panel are typical immunoblots showing the densitometric changes of each agent according to treatments vs. untreated controls (CTR). LC, loading control. The graphs underneath show the densitometric evaluations of the specific bands at each time point of every treatment. Points on the curves are mean values of three independent experiments, each carried out in duplicate with control (0-h) values normalized as 1.0. One-way *ANOVA* followed by post hoc Tukey’s test allowed to calculate *p* values. * *p* < 0.05 vs. CTR; ** *p* < 0.05 vs. fAβ_25–35_ + NPS 2143.

Densitometric analysis of immunoblots specific bands showed that after a 24 h delay the lysates’ IL-6 levels rose significantly (+90.1% by 48 h; + 77.3% by 72 h; *p* < 0.05 vs. 0 h control levels in both instances) in fAβ_25–__35_-treated NAHAs. Conversely, the NPS 2143 + fAβ_25–35_ treatment promptly increased the lysates’ IL-6 amounts (24 h, +111%; 48 h, +101%; 72 h, +73.3%; *p* < 0.05 vs. 0 h levels in all instances) (Figure 4A). Thus, by hindering extracellular release, NPS 2143 treatment favored an early intracellular accumulation of IL-6 which concurred with a decrease in IL-6 secretion (cf. Figure 3A).

#### 3.3.2. ICAM-1 Holoprotein Expression in NAHA Protein Lysates

The exposure to fAβ_25–35_ alone increased in a linear fashion over basal levels the ICAM-1 holoprotein contents of NAHAs lysates (24 h, +267%; 48 h, +600%; 72 h, +1061%; *p* < 0.001 at all time points examined) (Figure 4B). In NPS, 2143 + fAβ_25–35_ co-exposed NAHAs the intracellular ICAM-1 holoprotein levels speedily rose between 0 h and 24 h (+600%; *p* < 0.001) to keep raising more slowly thereafter (48 h, +700%; 72 h, +760%; *p* < 0.001 in both instances). Thus, fAβ_25–__35_ by itself remarkably increased the intracellular accumulation of the ICAM-1 holoprotein. The NPS 2143 addition only intensified the ICAM-1 holoprotein accumulation during the first 24 h while reducing it by 72 h vs. fAβ_25–__35_ alone values. This happened just while the s-ICAM-1 fragment shedding significantly decreased or fell to basal levels (cf. Figure 3B). At variance with the three other agents investigated, NPS 2143 addition likely promoted the ICAM-1 holoprotein intracellular proteolysis instead of its accumulation, while hindering the s-ICAM-1 fragment extracellular shedding.

#### 3.3.3. RANTES Expression in NAHAs Protein Lysates

In protein lysates from NAHAs exposed to fAβ_25–35_ by itself RANTES levels were lower than basal (0-h) values (24 h, −43%; 48 h, −57%; *p* < 0.05 at both time-points), to raise over basal values by 72 h (+36%; *p* < 0.05). Adding NPS 2143 to fAβ_25–__35_ initially increased RANTES levels in NAHAs lysates over basal (0-h) values (24 h, +93%; 48 h, +43%; *p* < 0.05 in both instances), but reduced them below basal values at 72 h (−29%; *p* < 0.05) (Figure 4C). Thus, the NPS 2143-antagonized Aβ•CaSR signaling favored the intracellular accumulation of RANTES while partially lessening its secretion (cf. Figure 3C).

#### 3.3.4. MCP-2 Expression in NAHA Protein Lysates

In lysates from fAβ_25–35_ alone-exposed NAHAs, MCP-2 protein was significantly upregulated at 24-h (+267%, *p* < 0.001 vs. basal [0-h] values), to decline thereafter yet remaining higher than basal levels (48 h, +100%; 72 h, +67%; *p* < 0.05 at both time-points). Adding NPS 2143 to fAβ_25–__35_-treatment linearly increased the MCP-2 contents (24 h, +156%; 48 h, +311%; 72 h, +556%; *p* < 0.001 at all time-points vs. basal [0 h] levels) in NAHA lysates (Figure 4D). Thus, by hindering Aβ•CaSR signaling NPS 2143 addition promoted the intracellular accumulation of MCP-2 while significantly hindering its secretion (cf. Figure 3D).

## 4. Discussion

**Figure 5:**
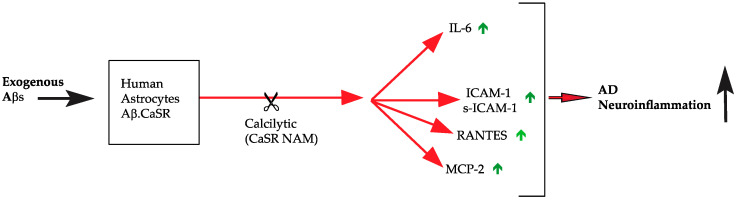
Synopsis of the direct pro-neuroinflammatory effects pathological Aβ•CaSR signaling evokes in NAHAs. The administration of a CaSR negative allosteric modulators (NAM) nearly totally switches off (in the case of IL-6) or remarkably mitigates (in the case of MCP-2, s-ICAM-1, and RANTES) the Aβ•CaSR signaling-evoked noxious pro-neuroinflammatory upshots. “**↑**” denotes upregulation. Abbreviations as in the text.

The present findings offer the first evidence ever that Aβ•CaSR signaling drives an increased synthesis and extracellular secretion/shedding of four proinflammatory agents, i.e., IL-6, ICAM-1/s-ICAM-1, RANTES, and MCP-2, from proliferatively quiescent cortical NAHAs (Figure 5). These results add a further noxious dimension to the previously reported multiple damaging effects that the pathological signaling from Aβ•CaSR complexes elicits in cultured NAHAs [18,19,20,21,35,36,39,54]. Most important, our results also show that a paradigmatic CaSR NAM, i.e., NPS 2143, can significantly suppress or abate NAHAs’ secretion/shedding of the same four proinflammatory mediators. Abundant data in the literature prove that these same agents concur to evoke significant neuroinflammatory effects in vivo (cf. Table 3). Activated astrocytes and microglia, the brain’s innate immune system cells, partake in neuroinflammation by producing and releasing copious amounts of cytokines, chemokines, and other agents [50,51,52,53,66,67]. Chemokine-attracted circulating immune cells cross a dysfunctional BBB and release proinflammatory agents intensifying the CNS tissue damage (see for reviews [2,3]). As the abundant literature on the topic makes clear (see for reviews [3,68]), the main mechanisms of inflammatory signaling develop as an integrated pattern forming a biological signaling network. In fact, once cytokines are secreted in response to initial signals, they can bind to their own receptors and trigger both cytosolic and nuclear signal amplification pathways (i.e., NF-κB, JNK [c-Jun N-terminal kinase), p38 MAPK (mitogen activated protein kinase), STAT (signal transducers and activators of transcription), and PI3K (phosphatidylinositol-3-kinase) that crosstalk with one another resulting in complex intracellular signaling networks. Reciprocal astrocytes-microglia interactions [9] lead to self-strengthening positive feedback loops perpetuating and spreading the neuroinflammation. Reportedly, IL-1β, IL-6, TNF-α, and IFN-γ are the main pro-inflammatory cytokines involved in AD brains [51,66,67,69]. Several chemokines, e.g., MCP-1 (or CCL2), RANTES (or CCL5), CCL23, IL-8 (or CXCL8), and IP-10 (or CXCL10) are involved too as they recruit peripheral immune cells into the CNS [51,67,69]. Neuroinflammation exacerbates the course of both acute (e.g., stroke) and chronic (e.g., AD, Parkinson, etc.) diseases by self-propagation and by causing neuronal excitotoxicity and loss of synapses [9,50,51,52,53,66]. Regarding AD, some reports suggest that microglial inflammatory mediators like IFN-γ and TNF-α cause Aβs overproduction and deposition by hindering Aβs clearance in mutant APP transgenic mice and in cocultures of astrocytes and microglia from the same mutant and wild-type mice [70,71]. At variance with these results in rodents, microglial cytokines only transiently accelerated endogenous Aβs release from Aβ-exposed NAHAs [21]. The use of antibody arrays has allowed us to find the four proinflammatory agents that are driven by Aβ•CaSR signaling in NAHAs and are hindered by the CaSR NAM presently employed.

### 4.1. IL-6

The present findings show that Aβ•CaSR signaling strongly increases both the synthesis and release of IL-6 from NAHAs. Conversely, calcilytic NPS 2143 suppresses most of this IL-6 surplus release while increasing its amounts in NAHAs lysates. The latter finding might be ascribed to the blocking effect of the IL-6 secretory pathway at the Golgi apparatus level [72] that CaSR NAM NPS 2143 exerts, which affects the secretion of other compounds including endogenous Aβ_42_ [20] and likely also RANTES and MCP-2 (see below). Further studies will clarify whether the slow decline in intracellular levels of IL-6 occurring after early peaking is due to decreased synthesis or increased lysis or both. It is well established that IL-6 and its downstream JAK/STAT3 signaling pathway exert pleiotropic effects closely related to our previous and present findings, that is the upregulation of the transcription of several genes including *ICAM-1*, *RANTES*, *MCP-2*, *VEGF-A*, and *CASR* [73,74,75]. On this basis, we posit that IL-6 overexpression driven by Aβ•CaSR signaling partakes in the control of *CASR*, *ICAM-1/s-ICAM-1*, *RANTES*, and *MCP-2* genes upregulation in NAHAs. Moreover, overproduced IL-6 accumulates around and inside senile plaques in the cerebral cortex and hippocampi of AD patients [76,77]. IL-6 also increases Tau protein hyperphosphorylation in neurons of AD brains through the cdk5/p35 and the MAPK-p38 signaling pathways [78]. Conversely, the results of studies about IL-6 in AD-model animals have so far been contradictory. In the astrocytes residing in the hippocampus and cerebellum of transgenic mice, increased IL-6 levels upregulated the expression of GFAP, glutamine synthase, STAT-3, phosphorylated STAT-3, and phosphorylated pp42/44 MAPK, but downregulated that of Synuclein 1, GAD65/67, GluA1, and GluN1 [79]. Recent reports revealed that the JAK/STAT3 pathway acts as pivotal driver of astrocyte reactivity [80] and that inhibiting the Stat3-mediated astrogliosis ameliorates the neuropathology in mouse models of AD [81]. Brugg et al. [82] reported that peripheral stimulation with LPS induced transient elevations in both IL-6 and IL-1β mRNAs followed by changes in the expression pattern of APP isoforms (i.e., decreases in APP695 and increases in APP KPI levels) in the cerebellum but not in the cerebral cortex of mouse brain. This concurrent upregulation of both IL-6 and APP during acute neurological stress or chronic neurodegeneration suggested an interlinked expression of these two proteins. However, as a cautionary note, we must mention here that NAHAs are insensitive to LPS exposure (our unpublished results) and, as the present findings show, IL-6 upregulation is completely or nearly completely controlled by Aβ•CaSR signaling in NAHAs.

### 4.2. ICAM-1 Holoprotein/s-ICAM-1

The present results show that the exposure to exogenous Aβs progressively raised ICAM-1 holoprotein’s levels in NAHAs lysates and, after a 24-h delay, even s-ICAM-1 fragment’s levels in NAHA-conditioned media. They also reveal for the first time that by antagonizing Aβ•CaSR signaling calcilytic NPS 2143 deeply (i.e., by about 64%; Table 2) cut down s-ICAM-1 fragment’s extracellular shedding from NAHAs while prompting an early and persistent increase of ICAM-1 holoprotein amounts in NAHA lysates. It is well known that cell adhesion molecules, including ICAM-1, regulate both physiological interactions between neural cells and the extracellular environment and pathological mechanisms underlying neurodegenerative diseases (reviewed in [83,84]). Belonging to the immunoglobulin (Ig) supergene family, the heavily N-glycosylated ICAM-1 (CD54) is an (Ig)-like holoprotein (~100 kDa) expressed by activated GFAP^+^ astrocytes and microglia both in the CNS grey and white matter and in vitro. ICAM-1′s Ig domains bind several leukocytes’ ligands including CD11a/CD18 (LFA-1), CD11b/CD18 (Mac-1), β_2_-integrins (p150,95 and Mac-1), and more. The binding of ligands to ICAM-1 homodimers’ [85] activates Akt, ERK, and JNK signaling pathways regulating (i) the synthesis of cytokines, chemokines, and adhesion molecules; and (ii) leukocytes’ traffic across the BBB (see references in [86]). Proteolysis of the membrane-inserted ICAM-1 ectodomain by cathepsin G, neutrophil elastase, and ADAM-17/TACE sheds the soluble s-ICAM-1 fragment (85 kDa) into the extracellular space, circulating blood, and cerebrospinal fluid (CSF) [86,87,88]. Microglial cytokines (e.g., IFN-β1a, IFN-γ, and TNF-α) and *Staphylococcus* enterotoxin B regulate s-ICAM-1 fragment’s shedding from endothelial cells, peripheral monocytes [87,89], and human cortical fetal astrocytes [64]. Sloughed off s-ICAM-1 fragment binds the same ligands as transmembrane ICAM-1 holoprotein does to activate leukocytes [90]. s-ICAM-1 also prevents leukocytes from binding membrane-inserted ICAM-1 holoprotein’s ectodomain [88]. Under physiological conditions, the basal expression of both ICAM-1 holoprotein and s-ICAM-1 fragment is weak and mostly occurs in endothelial cells. Various cytokines, including IL-6, RANTES, IL-1β, TNF-α, and IFN-γ upregulate the expression of both ICAM-1 holoprotein and s-ICAM-1 fragment, which in turn actively drive inflammatory responses and hence are inflammation markers [64,89,90,91,92,93]. Studies comparing s-ICAM-1′s with soluble E selectin’s expression levels in AD brains indicated that s-ICAM-1 fragment surpluses arise from neural cells (likely astrocytes) rather than from endothelial cells, since soluble E selectin levels, an exclusive marker of endothelial cells activation, remained unchanged [94].

Based on the just mentioned data in the literature, our findings suggest that by increasing both IL-6 and RANTES expression Aβ•CaSR signaling boosted a sizeable chunk of s-ICAM-1 fragment’s shedding from NAHAs. The calcilytic-non-suppressible portion of s-ICAM-1′s increased shedding might have been brought about by the concurrently Aβ-elicited IL-1β upregulation [86,87,95], which as our results show, is independent of Aβ•CaSR signaling. Further studies will assess this hypothesis. Conversely, the observed ICAM-1 holoprotein increases in NAHA lysates are likely to be due to different operative mechanisms. On its own part, Aβ•CaSR signaling might increase ICAM-1 holoprotein synthesis and its insertion and cleavage at the NAHAs plasma membrane. On the other hand, the calcilytic might hinder plasma the ICAM-1 holoprotein membrane insertion and cleavage. Although the details about the molecular mechanisms involved are not understood, our results clearly show that the addition of NPS 2143 to Aβ-exposed NAHAs did significantly cut down the potential proinflammatory actions of both ICAM-1 holoprotein and s-ICAM-1 fragment. The following evidences will allow us to better appreciate the prospective relevance of these findings. Remarkable increases in extravascular s-ICAM-1 around GFAP^+^ astrocytes connote the orbitofrontal cortex of normally aging people and might mark an increasing with age risk of neuroinflammatory diseases [88]. In postmortem AD brains, s-ICAM-1 aggregates localize to peri-plaque astrocytes, early and late stage amyloid senile plaques, and cerebral vessels [96,97,98]. Serum levels of s-ICAM-1 are low in healthy subjects [89]. In AD cases, s-ICAM-1 levels raise in both blood and CSF, mirror the upsurges in transmembrane ICAM-1 holoprotein, positively correlate with illness severity [90,91,95], and partake in BBB’s dysfunction thus advancing immune cells infiltration into the CNS and hence neuroinflammation.

### 4.3. RANTES

Astrocytes are the main source of RANTES, a powerful chemokine that attracts and activates eosinophil and basophil leukocytes. The present results show that, after a 24 h delay, Aβ-exposed NAHAs released significantly higher RANTES amounts while reducing its levels in the cells’ lysates. The 24 h time lag preceding the onset of RANTES over-release suggests that its driving mechanism(s) is(are) complex requiring the synthesis of some intermediate agent(s). Microglial cytokines like TNF-α and IFN-γ upregulate RANTES expression in human and simian astrocytes [66]. However, these cytokines were undetectable in our experimental system. Of greater interest is the notion that IL-1β mediates RANTES expression in human fetal astrocytes via the activation of IFN regulatory factor 3 (IRF3]. In its turn, IFR3 induces a group of IFN-stimulated antiviral response genes (ISG) including, besides RANTES, IFN-β, IRF7, and CXCL10/IFN-γ-inducible protein-10 [62,99]. Whether this IL-1β-triggered mechanism also works in the Aβ-treated NAHAs that, as our results show, overexpress IL-1β (Figure S1), seems likely yet remains to be proven. Lin et al. [100] also reported that the activation of PI3K and MAPK signaling pathways upregulated RANTES expression in curcumin-treated primary rat astrocytes. Because Aβ•CaSR signaling activates MEK/ERK signaling in NAHAs [101], this mechanism could also help up-regulate RANTES. This view is supported by our observation that CaSR NAM NPS 2143 significantly curbed a substantial fraction (i.e., 42%; Table 2) of RANTES release from NAHAs likely via a block of its secretory pathway through the Golgi apparatus—a mechanism also shared by IL-6, MCP-2, and endogenous Aβ_42_ [21,102,103]. On the other hand, the fall of immunodetectable RANTES in lysates from Aβ-exposed NAHAs suggests that over synthesized RANTES is secreted into the medium with no delay just as happens for VEGF-A [35]. Moreover, our observation that after peaking at 24-h the RANTES overaccumulation progressively vanished in Aβ+NPS 2143-treated NAHAs might result from (i) an initial block of RANTES secretory pathway through the Golgi apparatus; and (ii) a later decline of RANTES de novo synthesis coupled to a rescue of proteasomal activity [21]. Clearly, a detailed definition of the mechanisms involved requires further investigations. Our results suggest as likely that RANTES over-release from Aβ-exposed NAHAs partakes in the multiple proinflammatory effects evoked by Aβ•CaSR signaling and by an Aβ exposure in general, which is in keeping with the views of authors positing that RANTES is a relevant player in the inflammatory cascade that advances AD neurodegeneration [62,104,105]. As it does in murine astrocytes, RANTES by itself also strongly stimulates the production and release of proinflammatory mediators, including s-ICAM-1 [94,106]. It is worth mentioning here the interactions of secreted RANTES with its three receptors, i.e., CCR1, CCR3, and CCR5, which drive autocrine mechanisms affecting astrocytes secretory functions [106]. Of note, the activation of RANTES receptors entails their coupling with inhibitory G_i/o_ protein, which cuts down adenylyl cyclase activity and thus lowers the synthesis of neurotrophic cyclic 3′,5′-adenosine monophosphate (cAMP) in mouse astrocytes [93]. Previously, we showed that CaSR NAM NPS 2143 rescued the Aβ-curtailed cAMP production and release from NAHAs [21]. Whether the signaling pathways of CASR and RANTES receptors crosstalk between each other is to be determined in NAHAs. Finally, on a discordant note, Grammas et al. [105,107] reported that RANTES upregulation assists neurons’ survival in vitro by protecting them against the noxious effects of AD neurotoxins, thrombin, and sodium nitroprusside. Therefore, further studies will clarify whether RANTES plays a double-face role according to the actual stage of AD neuroinflammation.

### 4.4. MCP-2

Human MCP-2 is a small chemokine encoded by the *CCL8* gene. MCP-2 pertains to a subfamily of the C-C or β-chemokines also including MCP-1 and MCP-3 sharing a 60–62% sequence identity [61]. The present findings prove for the first time that Aβ-exposed NAHAs quite intensely oversecrete MCP-2 with respect to basal values. They also show that adding calcilytic NPS 2143 cuts down a significant fraction (62.3%; Table 2) of the Aβ•CaSR signaling-driven MCP-2 oversecretion. MCP-2 only moderately accumulated in lysates from Aβ-treated NAHAs apparently because its oversecretion balanced most of its overproduction. However, the linearly progressive and much more pronounced MCP-2 accumulation in lysates from Aβ+NPS 2143-treated NAHAs suggests that NPS 2143 blocked MCP-2 secretion through the Golgi pathway, as did with IL-6, RANTES, and endogenous Aβ_42_ [21,102,103], without interfering with its accelerated synthesis. Further work will assess this postulation. As a chemokine, MCP-2 is both less potent and less effective than MCP-1, MCP-3, and RANTES [61,108]. But, as our findings show, basal MCP-1 expression did not change in Aβ-treated NAHAs (Figure S1). Reportedly, MCP-2 activates the chemotaxis of human lymphocytes T, NK cells, and monocytes, which take part in inflammatory responses, and of eosinophils, basophils, and mast cells, which partake in allergic reactions. Concerning AD, most of the existing literature focuses on the proinflammatory role of MCP-1. Conversely, the available data about MCP-2 role(s) in AD are scanty. Just like MCP-1, CCL7, CCL12, and CCL13, MCP-2 enhances the chemotaxis of proinflammatory cells towards inflamed areas of the CNS [109]. As happens with MCP-1, CSF MCP-2 levels might increase in AD patients, suggesting its association with neurodegeneration [108]. In addition, CSF levels of MCP-2 might be a good risk predictive marker for early stage AD and other psychoses [110]. Aβ•CaSR signaling and other hitherto unidentified factors may drive MCP-2 oversecretion from NAHAs. Next, MCP-2 would recruit monocytes and/or other leukocyte populations into the brain, thereby enhancing neuroinflammation and neuronal injury, thus contributing to AD’s progression via mechanisms not detectable by the presently used experimental system. Based on our findings, it seems workable that by antagonizing Aβ•CaSR signaling, calcilytics could significantly diminish MCP-2 role in AD’s neuroinflammation. Clearly, further studies should specifically address and clarify MCP-2′s role(s) in human AD.

### 4.5. Other Proinflammatory Agents Not Affected by Antagonizing Aβ•CaSR Signaling

See Supplementary Data.

## 5. Conclusions

The present results are the first evidence that Aβ•CaSR signaling is directly involved in AD’s neuroinflammation via the over-release/shedding of four proinflammatory agents from NAHAs. This is a further addition to the previously reported panoply of detrimental actions driven by the Aβ•CaSR signaling in NAHAs and human neurons [14,17,18,19,20,33,34,52]. Notably, the Aβ•CaSR signaling unique ability to simultaneously set off and release an amazing multiplicity of noxious effectors from human cortical neurons and astrocytes, testifies for its relevance to AD. Our findings further stress the view that pathological Aβ•CaSR signaling is a potential therapeutic target in AD [18,20,21,36,37,38]. On the other hand, they also show that extracellular Aβs surpluses induce NAHAs to over-release several proinflammatory agents through Aβ•CaSR-independent mechanisms. But these effects are placed downstream from Aβ•CaSR signaling, the upshots of which include the release of Aβs surpluses from human neurons and astrocytes [21]. As a final notation, we are aware that the other outstanding neuroinflammation player, i.e., human microglia, is missing from the experimental system presently used. However, we are confident that future work will overcome this limitation and throw further light onto the intricate mechanisms that hold sway in human AD-related neuroinflammation

## Figures and Tables

**Table 1:** Proinflammatory agents analyzed via antibody array in conditioned media of untreated NAHAs.

Cytokines			
Name	Gene	UniProtKB ID	Expression
Interleukin-1α	*IL1A*	P01583	nd
Interleukin-1β	*IL1B*	P01584	+
Interleukin-2	*IL2*	P60568	nd
Interleukin-3	*IL3*	P08700	+
Interleukin-4	*IL4*	P05112	nd
Interleukin-6	*IL6*	P05231	+
Interleukin-7	*IL7*	P13232	nd
Interleukin-10	*IL10*	P22301	nd
Interleukin-11	*IL11*	P20809	nd
Interleukin-12 subunit-α	*IL12A*	P29459	nd
Interleukin-12 subunit-β	*IL12B*	P29460	nd
Interleukin-13	*IL13*	P35225	nd
Interleukin-15	*IL15*	P40933	nd
Interleukin-16	*IL16*	Q14005	+
Interleukin-17A	*IL17*	Q16552	nd
Interferon-γ	*IFNG*	P01579	nd
Granulocyte colony-stimulating factor	*CSF3*	P09919	nd
Granulocyte-macrophage colony-stimulating factor	*CSF2*	P04141	nd
Lymphotoxin-α	*TNFB*	P01374	nd
Macrophage colony-stimulating factor-1	*CSF1*	P09603	nd
Transforming growth factor-β1	*TGFB1*	P01137	nd
Tumor necrosis factor-α	*TNFA*	P01375	nd
Cytokine Soluble Receptors			
Interleukin-6 receptor subunit-α	*IL6R*	P08887	nd
TNF receptor superfamily member-1α	*TNFRSF1A*	P19438	nd
TNF receptor Superfamily Member-1β	*TNFRSF1B*	P20333	nd
Chemokines			
C-C motif chemokine-1	*CCL1*	P22362	nd
C-C motif chemokine-5/RANTES	*CCL5*	P13501	+
C-X-C motif chemokine-9	*CXCL9*	Q07325	nd
C-X-C motif chemokine-10	*CXCL10*	P02778	nd
Eotaxin-1	*CCL11*	P51671	nd
Eotaxin-2	*CCL24*	O00175	nd
Interleukin-8	*CXCL8*	P10145	+
Monocyte chemoattractant protein-1	*CCL2/MCP1*	P13500	+
Monocyte chemoattractant protein-2	*CCL8/MCP2*	P80075	+
Macrophage inflammatory protein-1α	*CCL3*	P10147	nd
Macrophage inflammatory protein-1β	*CCL4*	P13236	nd
Macrophage inflammatory protein-5	*CCL15*	Q16663	nd
Other agents			
Soluble intercellular adhesion molecule-1 (s-ICAM-1]	*ICAM1*	P05362	+
Metalloproteinase inhibitor-2	*TIMP2*	P16035	+
Platelet-derived growth factor subunit-B	*PDGFB*	P01127	+

nd, not detected; +, detected.

**Table 2:** Impact of calcilytic NPS 2143 on fAβ_25–35_-evoked cumulative 0-h-to-96-h secretion/shedding of neuroinflammatory agents from NAHAs.

	Treatments	Cumulative Secretion ^§^	% Changes vs. CTR	% Changes vs. fAβ25–35
IL-6	*CTR*	502.1 ± 39.6		
	*fAβ_25–35_*	905.6 ± 48.4	+80.4 *	
	*fAβ_25–35_ +NPS 2143*	576.9 ± 41.0	+14.9	−81.5 *
s-ICAM-1	*CTR*	30.6 ± 2.3		
	*fAβ_25–35_*	59.7 ± 2.7	+95.1 *	
	*fAβ_25–35_ + NPS 2143*	41 ± 2.6	+34.0 *	−64.3 *
RANTES	*CTR*	42.9 ± 13.3		
	*fAβ_25–35_*	226 ± 17.2	+426.8 *	
	*fAβ_25–35_ + NPS 2143*	149.3 ± 7.4	+248.0 *	−41.9 *
MCP-2	*CTR*	185.9 ± 29.3		
	*fAβ_25–35_*	6897.9 ± 248	+3611.0 *	
	*fAβ_25–35_ + NPS 2143*	2717.6 ± 320	+1361.9 *	−62.3 *

^§^ Expressed as mean values ± SEM of the areas under the respective 0 h-to-96 h curves. *, *p* < 0.05. CTR, untreated controls.

**Table 3:** IL-6, s-ICAM-1, RANTES, and MCP-2 drive complex neuroinflammatory responses.

Agent	Proinflammatory Roles
IL-6	Induces extensive gliosis and microglial phagocytosis of Aβs deposits in vivo [77] Increases Tau protein hyperphosphorylation in neurons [78] Increases the levels of hippocampal and cerebellar GFAP, glutamine synthase, STAT-3, phosphorylated STAT-3, and phosphorylated pp42/44 MAPK [79] Decreases the levels of Syn 1, GAD65/67, GluA1, and GluN1 [79] Upregulates *CASR* gene transcription [75] Upregulates the transcription of several other genes including *ICAM-1, RANTES, MCP-2, VEGF-A* [73,74]
s-ICAM-1	Promotes lymphocytes and leukocyte trafficking through the BBB [87] Partakes in BBB dysfunction and CNS infiltration by immune cells [87] Localizes to vessels, early and late stage amyloid senile plaques, and peri-plaque astrocytes in AD brains [96,97]
RANTES	Attracts and activates eosinophil and basophil leukocytes [104] Is relevant to the neuroinflammatory cascade that contributes to neurodegeneration in AD brains [62,104] Stimulates astrocytes’ production and release of proinflammatory mediators [93,106] The RANTES/CCR3 signaling lowers the endogenous levels of cAMP [106] May contribute to upregulating the s-ICAM-1 levels [93]
MCP-2	Enhances chemotaxis of proinflammatory cells (monocytes or other leukocyte populations) to inflamed CNS areas [109]

## Supplementary Materials

The following are available online at https://www.mdpi.com/2073-4409/9/6/1386/s1, Figure S1: Other Cytokines and Chemokines Arrays, Figure S2: PDGF-BB Array.

## References

[1] Prince M.J., Wimo A., Guerchet M.M., Ali G.C., Wu Y.-T., Prina M. (2015). World Alzheimer Report 2015: The Global Impact of Dementia: An Analysis of Prevalence, Incidence, Cost and Trends.

[2] Labzin L.I., Heneka M.T., Latz E. (2018). Innate immunity and neurodegeneration. Annu. Rev. Med..

[3] Becher B., Spath S., Goverman J. (2017). Cytokine networks in neuroinflammation. Nat. Rev. Immunol..

[4] Hardy J., Selkoe D.J. (2002). The amyloid hypothesis of Alzheimer’s disease: Progress and problems on the road to therapeutics. Science.

[5] Braak H., Del Tredici K. (2015). The preclinical phase of the pathological process underlying sporadic Alzheimer’s disease. Brain.

[6] Medeiros R., LaFerla F.M. (2013). Astrocytes: Conductors of the Alzheimer disease neuroinflammatory symphony. Exp. Neurol..

[7] Rodríguez-Arellano J.J., Parpura V., Zorec R., Verkhratsky A. (2016). Astrocytes in physiological aging and Alzheimer’s disease. Neuroscience.

[8] Kato S., Gondo T., Hoshii Y., Takahashi M., Yamada M., Ishihara T. (1998). Confocal observation of senile plaques in Alzheimer’s disease: Senile plaque morphology and relationship between senile plaques and astrocytes. Pathol. Int..

[9] Von Bernhardi R., Ramirez G. (2001). Microglia-astrocyte interaction in Alzheimer’s disease: Friends or foes for the nervous system?. Biol. Res..

[10] Oberheim N.A., Takano T., Han X., He W., Lin J.H., Wang F., Xu Q., Wyatt J.D., Pilcher W., Ojemann J.G. (2009). Uniquely hominid features of adult human astrocytes. J. Neurosci..

[11] Hodge R.D., Bakken T.E., Miller J.A., Smith K.A., Barkan E.R., Graybuck L.T., Close J.L., Long B., Johansen N., Penn O. (2019). Conserved cell types with divergent features in human versus mouse cortex. Nature.

[12] Ullian E.M., Sapperstein S.K., Christopherson K.S., Barres B.A. (2001). Control of synapse number by glia. Science.

[13] Goodall E.F., Wang C., Simpson J.E., Baker D.J., Drew D.R., Heath P.R., Saffrey M.J., Romero I.A., Wharton S.B. (2018). Age-associated changes in the blood-brain barrier: Comparative studies in human and mouse. Neuropathol. Appl. Neurobiol..

[14] Zhang Y., Sloan S.A., Clarke L.E., Caneda C., Plaza C.A., Blumenthal P.D., Vogel H., Steinberg G.K., Edwards M.S., Li G. (2016). Purification and Characterization of Progenitor and Mature Human Astrocytes Reveals Transcriptional and Functional Differences with Mouse. Neuron.

[15] Dal Prà I., Chiarini A., Pacchiana R., Gardenal E., Chakravarthy B., Whitfield J.F., Armato U. (2014). Calcium-Sensing Receptors of Human Astrocyte-Neuron Teams: Amyloid-β-Driven Mediators and Therapeutic Targets of Alzheimer’s Disease. Curr. Neuropharmacol..

[16] Cummings J.L., Morstorf T., Zhong K. (2014). Alzheimer’s disease drug-development pipeline: Few candidates, frequent failures. Alzheimers Res. Ther..

[17] Drummond E., Wisniewski T. (2017). Alzheimer’s disease: Experimental models and reality. Acta Neuropathol..

[18] Chiarini A., Armato U., Liu D., Dal Prà I. (2016). Calcium-Sensing Receptors of Human Neural Cells Play Crucial Roles in Alzheimer’s Disease. Front. Physiol..

[19] Dal Prà I., Armato U., Chiarini A. (2019). Family C G-Protein-Coupled Receptors in Alzheimer’s Disease and Therapeutic Implications. Front. Pharmacol..

[20] Armato U., Chiarini A., Chakravarthy B., Chioffi F., Pacchiana R., Colarusso E., Whitfield J.F., Dal Prà I. (2013). Calcium-sensing receptor antagonist (calcilytic) NPS 2143 specifically blocks the increased secretion of endogenous Aβ42 prompted by exogenous fibrillary or soluble Aβ25–35 in human cortical astrocytes and neurons¾Therapeutic relevance to Alzheimer’s disease. Biochim. Biophys. Acta.

[21] Chiarini A., Armato U., Liu D., Dal Prà I. (2017). Calcium-Sensing Receptor Antagonist NPS 2143 Restores Amyloid Precursor Protein Physiological Non-Amyloidogenic Processing in Aβ-Exposed Adult Human Astrocytes. Sci. Rep..

[22] Nemeth E.F., Goodman W.G. (2016). Calcimimetic and calcilytic drugs: Feats, flops, and futures. Calcif. Tissue Int..

[23] Zhang C., Miller C.L., Brown E.M., Yang J.J. (2015). The calcium sensing receptor: From calcium sensing to signaling. Sci. China Life Sci..

[24] Chakravarty B., Chattopadhyay N., Brown E.M. (2012). Signaling through the extracellular calcium-sensing receptor (CaSR). Adv. Exp. Med. Biol..

[25] Bandyopadhyay S., Tfelt-Hansen J., Chattopadhyay N. (2010). Diverse roles of extracellular calcium-sensing receptor in the central nervous system. J. Neurosci. Res..

[26] Chattopadhyay N., Evliyaoglu C., Heese O., Carroll R., Sanders J., Black P., Brown E.M. (2000). Regulation of secretion of PTHrP by Ca^2+^-sensing receptor in human astrocytes, astrocytomas, and meningiomas. Am. J. Physiol. Cell. Physiol..

[27] Dal Prà I., Chiarini A., Nemeth E.F., Armato U., Whitfield J.F. (2005). Roles of Ca^2+^ and the Ca^2+^-sensing receptor (CaSR) in the expression of inducible NOS (nitric oxide synthase)-2 and its BH4 (tetrahydrobiopterin)-dependent activation in cytokine-stimulated adult human astrocytes. J. Cell. Biochem..

[28] Hofer A.M., Brown E.M. (2003). Extracellular calcium sensing and signalling. Nat. Rev. Mol. Cell Biol..

[29] Guo Y., Yang X., He J., Liu J., Yang S., Dong H. (2018). Important roles of the Ca^2+^-sensing receptor in vascular health and disease. Life Sci..

[30] Conigrave A.D., Hampson D.R. (2006). Broad-spectrum L-amino acid sensing by class 3 G-protein-coupled receptors. Trends Endocrinol. Metab..

[31] Noh J.S., Pak H.J., Shin Y.J., Riew T.R., Park J.H., Moon Y.W., Lee M.Y. (2015). Differential expression of the calcium-sensing receptor in the ischemic and border zones after transient focal cerebral ischemia in rats. J. Chem. Neuroanat..

[32] Chattopadhyay N., Ye C., Yamaguchi T., Nakai M., Kifor O., Vassilev P.M., Nishimura R.N., Brown E.M. (1999). The extracellular calcium-sensing receptor is expressed in rat microglia and modulates an outward K+ channel. J. Neurochem..

[33] Riccardi D., Kemp P.J. (2012). The calcium-sensing receptor beyond extracellular calcium homeostasis: Conception, development, adult physiology, and disease. Annu. Rev. Physyiol..

[34] Ruat M., Traiffort E. (2013). Roles of the calcium sensing receptor in the central nervous system. Best Pract. Res. Clin. Endocrinol. Metab..

[35] Dal Prà I., Armato U., Chioffi F., Pacchiana R., Whitfield J.F., Chakravarthy B., Gui L., Chiarini A. (2014). The Aβ peptides-activated calcium-sensing receptor stimulates the production and secretion of vascular endothelial growth factor-A by normoxic adult human cortical astrocytes. Neuromolecular Med..

[36] Chiarini A., Armato U., Gardenal E., Gui L., Dal Prà I. (2017). Amyloid β-Exposed Human Astrocytes Overproduce Phospho-Tau and Overrelease It within Exosomes, Effects Suppressed by Calcilytic NPS 2143-Further Implications for Alzheimer’s Therapy. Front. Neurosci..

[37] Kim J.Y., Ho H., Kim N., Liu J., Yenari M.A., Wenhan C. (2014). Calcium-sensing receptor (CaSR): A novel target for ischemic neuroprotection. Ann. Clin. Transl. Neurol..

[38] Bai S., Mao M., Tian L., Yu Y., Zeng J., Ouyang K., Yu L., Li L., Wang D., Deng X. (2015). Calcium sensing receptor mediated the excessive generation of β-amyloid peptide induced by hypoxia in vivo and in vitro. Biochem. Biophys. Res. Commun..

[39] Gardenal E., Chiarini A., Armato U., Dal Prà I., Verkhratsky A., Rodríguez J.J. (2017). Increased Calcium-Sensing Receptor Immunoreactivity in the Hippocampus of a Triple Transgenic Mouse Model of Alzheimer’s Disease. Front. Neurosci..

[40] Klein G.L., Castro S.M., Garofalo R.P. (2016). The calcium-sensing receptor as a mediator of inflammation. Semin. Cell Dev. Biol..

[41] Yarova P.L., Stewart A.L., Sathish V., Britt R.D., Thompson M.A.P., Lowe A.P., Freeman M., Aravamudan B., Kita H., Brennan S.C. (2015). Calcium-sensing receptor antagonists abrogate airway hyperresponsiveness and inflammation in allergic asthma. Sci. Transl. Med..

[42] Mattar P., Bravo-Sagua R., Tobar N., Fuentes C., Troncoso R., Breitwieser G., Lavandero S., Cifuentes M. (2018). Autophagy mediates calcium-sensing receptor-induced TNFα production in human preadipocytes. Biochim. Biophys. Acta Mol. Basis Dis..

[43] Iamartino L., Elajnaf T., Kallay E., Schepelmann M. (2018). Calcium-sensing receptor in colorectal inflammation and cancer: Current insights and future perspectives. World J. Gastroenterol..

[44] Bernichtein S., Pigat N., Barry Delongchamps N., Boutillon F., Verkarre V., Camparo P., Reyes-Gomez E., Méjean A., Oudard S.M., Lepicard E.M. (2017). Vitamin D3 Prevents Calcium-Induced Progression of Early-Stage Prostate Tumors by Counteracting TRPC6 and Calcium Sensing Receptor Upregulation. Cancer Res..

[45] Lee J.W., Park H.A., Kwon O.K., Park J.W., Lee G., Lee H.J., Lee S.J., Oh S.R., Ahn K.S. (2017). NPS 2143, a selective calcium-sensing receptor antagonist inhibits lipopolysaccharide-induced pulmonary inflammation. Mol. Immunol..

[46] Hu B., Tong F., Xu L., Shen Z., Yan L., Xu G., Shen R. (2018). Role of Calcium Sensing Receptor in Streptozotocin-Induced Diabetic Rats Exposed to Renal Ischemia Reperfusion Injury. Kidney Blood Press. Res..

[47] Lau L.T., Yu A.C. (2001). Astrocytes produce and release interleukin-1, interleukin-6, tumor necrosis factor alpha and interferon-gamma following traumatic and metabolic injury. J. Neurotrauma.

[48] Benveniste E.N. (1998). Cytokine actions in the central nervous system. Cytokine Growth Factor Rev..

[49] Pratt B.M., McPherson J.M. (1997). TGF-beta in the central nervous system: Potential roles in ischemic injury and neurodegenerative diseases. Cytokine Growth Factor Rev..

[50] Parajuli B., Sonobe Y., Horiuchi H., Takeuchi H., Mizuno T., Suzumura A. (2013). Oligomeric amyloid beta induces IL-1beta processing via production of ROS: Implication in Alzheimer’s disease. Cell Death Dis..

[51] Ambrosini E., Remoli M.E., Giacomini E., Rosicarelli B., Serafini B., Lande R., Aloisi F., Coccia E.M. (2005). Astrocytes produce dendritic cell-attracting chemokines in vitro and in multiple sclerosis lesions. J. Neuropathol. Exp. Neurol..

[52] Strack A., Asensio V.C., Campbell I.L., Schlüter D., Deckert M. (2002). Chemokines are differentially expressed by astrocytes, microglia and inflammatory leukocytes in Toxoplasma encephalitis and critically regulated by interferon-gamma. Acta Neuropathol..

[53] Sokolova A., Hill M.D., Rahimi F., Warden L.A., Halliday G.M., Shepherd C.E. (2009). Monocyte chemoattractant protein-1 plays a dominant role in the chronic inflammation observed in Alzheimer’s disease. Brain Pathol..

[54] Chiarini A., Dal Pra I., Menapace L., Pacchiana R., Whitfield J.F., Armato U. (2005). Soluble amyloid beta-peptide and myelin basic protein strongly stimulate, alone and in synergism with joint proinflammatory cytokines, the expression of functional nitric oxide synthase-2 in normal adult human astrocytes. Int. J. Mol. Med..

[55] Dal Prà I., Armato U., Chiarini A. Specific interactions of calcium-sensing receptors (CaSRs) with soluble amyloid-β peptides—A study using cultured normofunctioning adult human astrocytes. Proceedings of the 2nd International Symposium on the Calcium-sensing Receptor.

[56] Gulyaeva N.V., Stepanichev M.Y. (2010). Abeta(25–35) as proxyholder for amyloidogenic peptides: In vivo evidence. Exp. Neurol..

[57] Zambrano A., Otth C., Mujica L., Concha I.I., Maccioni R.B. (2007). Interleukin-3 prevents neuronal death induced by amyloid peptide. BMC Neurosci..

[58] Di Rosa M., Dell’Ombra N., Zambito A.M., Malaguarnera M., Nicoletti F., Malaguarnera L. (2006). Chitotriosidase and inflammatory mediator levels in Alzheimer’s disease and cerebrovascular dementia. Eur. J. Neurosci..

[59] Ashutosh, Kou W., Cotter R., Borgmann K., Wu L., Persidsky R., Sakhuja N., Ghorpade A. (2011). CXCL8 protects human neurons from amyloid-beta-induced neurotoxicity: Relevance to Alzheimer’s disease. Biochem. Biophys. Res. Commun..

[60] Liu C., Cui G., Zhu M., Kang X., Guo H. (2014). Neuroinflammation in Alzheimer’s disease: Chemokines produced by astrocytes and chemokine receptors. Int. J. Clin. Exp. Pathol..

[61] Weber M., Uguccioni M., Ochensberger B., Baggiolini M., Clark-Lewis I., Dahinden C.A. (1995). Monocyte chemotactic protein MCP-2 activates human basophil and eosinophil leukocytes similar to MCP-3. J. Immunol..

[62] Kim M.O., Suh H.S., Brosnan C.F., Lee S.C. (2004). Regulation of RANTES/CCL5 expression in human astrocytes by interleukin-1 and interferon-beta. J. Neurochem..

[63] Lee E., Kim H. (2014). The anti-inflammatory role of tissue inhibitor of metalloproteinase-2 in lipopolysaccharide-stimulated microglia. J. Neuroinflammation.

[64] Lee S.J., Benveniste E.N. (1999). Adhesion molecule expression and regulation on cells of the central nervous system. J. Neuroimmunol..

[65] Zhou Z., Wu Q., Lu Y., Zhang X., Lv S., Shao J., Zhou Y., Chen J., Hou L., Huang C. (2019). Crosstalk between soluble PDGF-BB and PDGFR b promotes astrocytic activation and synaptic recovery in the hippocampus after subarachnoid hemorrhage. FASEB J..

[66] Croitoru-Lamoury J., Guillemin G.J., Boussin F.D., Mognetti B., Gigout L.I., Chéret A., Vaslin B., Le Grand R., Brew B.J., Dormont D. (2003). Expression of chemokines and their receptors in human and simian astrocytes: Evidence for a central role of TNF alpha and IFN gamma in CXCR4 and CCR5 modulation. Glia.

[67] Wang W.Y., Tan M.S., Yu J.T., Tan L. (2015). Role of pro-inflammatory cytokines released from microglia in Alzheimer’s disease. Ann. Transl. Med..

[68] Schmitz M.L., Weber A., Roxlau T., Gaestel M., Kracht M. (2011). Signal integration, crosstalk mechanisms and networks in the function of inflammatory cytokines. Biochim. Biophys. Acta.

[69] Kitazawa M., Cheng D., Tsukamoto M.R., Koike M.A., Wes P.D., Vasilevko V., Cribbs D.H., LaFerla F.M. (2011). Blocking IL-1 signaling rescues cognition, attenuates tau pathology, and restores neuronal β-catenin pathway function in an Alzheimer’s disease model. J. Immunol..

[70] Yamamoto M., Kiyota T., Horiba M., Buescher J.L., Walsh S.M., Gendelman H.E., Ikezu T. (2007). Interferon-gamma and tumor necrosis factor-alpha regulate amyloid-beta plaque deposition and beta-secretase expression in Swedish mutant APP transgenic mice. Am. J. Pathol..

[71] Zhao J., O’Connor T., Vassar R. (2011). The contribution of activated astrocytes to Aβ production: Implications for Alzheimer’s disease pathogenesis. J. Neuroinflammation.

[72] Tsakiri N., Kimber I., Rothwell N.J., Pinteaux E. (2008). Mechanisms of interleukin-6 synthesis and release induced by interleukin-1 and cell depolarisation in neurones. Mol. Cell. Neurosci..

[73] Yu H., Pardoll D., Jove R. (2009). STATs in cancer inflammation and immunity: A leading role for STAT3. Nat. Rev. Cancer.

[74] Wingender E. (2008). The TRANSFAC project as an example of framework technology that supports the analysis of genomic regulation. Brief Bioinform..

[75] Canaff L., Zhou X., Hendy G.N. (2008). The proinflammatory cytokine, interleukin-6, upregulates calcium-sensing receptor gene transcription via Stat 1/3 and Sp 1/3. J. Biol. Chem..

[76] Devaraj S., Glaser N., Griffen S., Wang-Polagruto J., Miguelino E., Jialal I. (2006). Increased monocytic activity and biomarkers of inflammation in patients with type 1 diabetes. Diabetes.

[77] Chakrabarty P., Jansen-West K., Beccard A., Ceballos-Diaz C., Levites Y., Verbeeck C., Zubair A.C., Dickson D., Golde T.E., Das P. (2010). Massive gliosis induced by interleukin-6 suppresses Abeta deposition in vivo: Evidence against inflammation as a driving force for amyloid deposition. FASEB J..

[78] Quintanilla R.A., Orellana D.I., González-Billault C., Maccioni R.B. (2004). Interleukin-6 induces Alzheimer-type phosphorylation of tau protein by deregulating the cdk5/p35 pathway. Exp. Cell Res..

[79] Gruol D.L., Vo K., Bray J.G. (2014). Increased astrocyte expression of IL-6 or CCL2 in transgenic mice alters levels of hippocampal and cerebellar proteins. Front. Cell. Neurosci..

[80] Haim L.B., Ceyzériat K., Carrillo-de Sauvage M.A., Aubry F., Auregan G., Guillermier M., Ruiz M., Petit F., Houitte D., Faivre E. (2015). The JAK/STAT3 Pathway Is a Common Inducer of Astrocyte Reactivity in Alzheimer’s and Huntington’s Diseases. J. Neurosci..

[81] Reichenbach N., Delekate A., Plescher M., Schmitt F., Krauss S., Blank N., Halle A., Petzold G.C. (2019). Inhibition of Stat3-mediated astrogliosis ameliorates pathology in an Alzheimer’s disease model. EMBO Mol. Med..

[82] Brugg B., Dubreuil Y.L., Huber G., Wollman E.E., Delhaye-Bouchaud N., Mariani J. (1995). Inflammatory processes induce beta-amyloid precursor protein changes in mouse brain. Proc. Natl. Acad. Sci. USA.

[83] Wennström M., Nielsen H.M. (2012). Cell adhesion molecules in Alzheimer’s disease. Degener. Neurol. Neuromuscul. Dis..

[84] Leshchyns’ka I., Sytnyk V. (2016). Synaptic Cell Adhesion Molecules in Alzheimer’s Disease. Neural. Plast..

[85] Staunton D.E., Marlin S.D., Stratowa C., Dustin M.L., Springer T.A. (1988). Primary structure of ICAM-1 demonstrates interaction between members of the immunoglobulin and integrin supergene families. Cell.

[86] Ramos T.N., Bullard D.C., Barnum S.R. (2014). ICAM-1: Isoforms and phenotypes. J. Immunol..

[87] Müller N. (2019). The Role of Intercellular Adhesion Molecule-1 in the Pathogenesis of Psychiatric Disorders. Front. Pharmacol..

[88] Tsakadze N.L., Sithu S.D., Sen U., English W.R., Murphy G., D’Souza S.E. (2006). Tumor necrosis factor-alpha-converting enzyme (TACE/ADAM-17) mediates the ectodomain cleavage of intercellular adhesion molecule-1 (ICAM- 1). J. Biol. Chem..

[89] Lawson C., Wolf S. (2009). ICAM-1 signaling in endothelial cells. Pharmacol. Rep..

[90] Schmal H., Czermak B.J., Lentsch A.B., Bless N.M., Beck-Schimmer B., Friedl H.P., Ward P.A. (1998). Soluble ICAM-1 activates lung macrophages and enhances lung injury. J. Immunol..

[91] Witkowska A.M., Borawska M.H. (2004). Soluble intercellular adhesion molecule-1 (s-ICAM-1): An overview. Eur. Cytokine Netw..

[92] Hua S. (2013). Targeting sites of inflammation: Intercellular adhesion molecule-1 as a target for novel inflammatory therapies. Front. Pharmacol..

[93] Luo Y., Berman M.A., Zhai Q., Fischer F.R., Abromson-Leeman S.R., Zhang Y., Kuziel W.A., Gerard C., Dorf M.E. (2002). RANTES stimulates inflammatory cascades and receptor modulation in murine astrocytes. Glia.

[94] Rentzos M., Michalopoulou M., Nikolaou C., Cambouri C., Rombos A., Dimitrakopoulos A., Vassilopoulos D. (2005). The role of soluble intercellular adhesion molecules in neurodegenerative disorders. J. Neurol. Sci..

[95] Miguel-Hidalgo J.J., Nithuairisg S., Stockmeier C., Rajkowska G. (2007). Distribution of ICAM-1 immunoreactivity during aging in the human orbitofrontal cortex. Brain Behav. Immun..

[96] Verbeek M.M., Otte-Höller I., Westphal J.R., Wesseling P., Ruiter D.J., de Waal R.M. (1994). Accumulation of intercellular adhesion molecule-1 in senile plaques in brain tissue of patients with Alzheimer’s disease. Am. J. Pathol..

[97] Akiyama H., Kawamata T., Yamada T., Tooyama I., Ishii T., McGeer P.L. (1993). Expression of intercellular adhesion molecule (ICAM)-1 by a subset of astrocytes in Alzheimer disease and some other degenerative neurological disorders. Acta Neuropathol..

[98] Walker D.G., Lue L.F., Tang T.M., Adler C.H., Caviness J.N., Sabbagh M.N., Serrano G.E., Sue L.I., Beach T.G. (2017). Changes in CD200 and intercellular adhesion molecule-1 (ICAM-1) levels in brains of Lewy body disorder cases are associated with amounts of Alzheimer’s pathology not alpha-synuclein pathology. Neurobiol. Aging.

[99] Rivieccio M.A., John G.R., Song X., Suh H.S., Zhao Y., Lee S.C., Brosnan C.F. (2005). The cytokine IL-1beta activates IFN response factor 3 in human fetal astrocytes in culture. J. Immunol..

[100] Lin M.S., Hung K.S., Chiu W.T., Sun Y.Y., Tsai S.H., Lin J.W., Lee Y.H. (2011). Curcumin enhances neuronal survival in N-methyl-d-aspartic acid toxicity by inducing RANTES expression in astrocytes via PI-3K and MAPK signaling pathways. Prog. Neuropsychopharmacol. Biol. Psychiatry.

[101] Chiarini A., Dal Pra I., Marconi M., Chakravarthy B., Whitfield J.F., Armato U. (2009). Calcium-sensing receptor (CaSR) in human brain’s pathophysiology: Roles in late-onset Alzheimer’s disease (LOAD). Curr. Pharm. Biotechnol..

[102] Chou S.Y., Weng J.Y., Lai H.L., Liao F., Sun S.H., Tu P.H., Dickson D.W., Chern Y. (2008). Expanded-polyglutamine huntingtin protein suppresses the secretion and production of a chemokine (CCL5/RANTES) by astrocytes. J. Neurosci..

[103] Stanley A.C., Lacy P. (2010). Pathways for cytokine secretion. Physiology (Bethesda).

[104] Appay V., Rowland-Jones S.L. (2001). RANTES: A versatile and controversial chemokine. Trends Immunol..

[105] Tripathy D., Thirumangalakudi L., Grammas P. (2010). RANTES upregulation in the Alzheimer’s disease brain: A possible neuroprotective role. Neurobiol Aging.

[106] Zhang Y., Luo Y., Zhai Q., Ma L., Dorf M.E. (2003). Negative role of cAMP-dependent protein kinase A in RANTES-mediated transcription of proinflammatory mediators through Raf. FASEB J..

[107] Sanchez A., Tripathy D., Grammas P. (2009). RANTES release contributes to the protective action of PACAP38 against sodium nitroprusside in cortical neurons. Neuropeptides.

[108] Galimberti D., Schoonenboom N., Scheltens P., Fenoglio C., Bouwman F., Venturelli E., Guidi I., Blankenstein M.A., Bresolin N., Scarpini E. (2006). Intrathecal chemokine synthesis in mild cognitive impairment and Alzheimer disease. Arch. Neurol..

[109] Stuart M.J., Singhal G., Baune B.T. (2015). Systematic Review of the Neurobiological Relevance of Chemokines to Psychiatric Disorders. Front. Cell. Neurosci..

[110] Hayes L.N., Severance E.G., Leek J.T., Gressitt K.L., Rohleder C., Coughlin J.M., Leweke F.M., Yolken R.H., Sawa A. (2014). Inflammatory molecular signature associated with infectious agents in psychosis. Schizophr. Bull..

