# Supplementary material for: CaSR Antagonist (Calcilytic) NPS 2143 Hinders the Release of Neuroinflammatory IL-6, Soluble ICAM-1, RANTES, and MCP-2 from Aβ-Exposed Human Cortical Astrocytes
Source: Cells. 2020 Jun 2;9(6):1386. doi: 10.3390/cells9061386 (PMC7349863; doi:10.3390/cells9061386)
Supplement: Supplementary file 1 [file cells-09-01386-s001.pdf]

S1. Supplementary Results and Discussion

S1.1. Other proinflammatory agents not affected by antagonizing Aβ•CaSR signaling

In keeping with previous works from other Laboratories [1-3], our results show that Aβ-exposed NAHAs overproduce and overrelease a set cytokines and chemokines, i.e. IL-1β, IL-3, IL-8, IL-16, the secretion of which NPS 2143 did not hinder [Table 1; Figure S1]. All these cytokines/chemokines have been linked to inflammatory brain disorders, and their functional roles are still under investigation. Notably, within this group IL-8 shows the highest basal expression in NAHAs-conditioned media as determined by the analysis of the antibody array, whereas IL-1β, IL-3, and IL-16 are nearly undetectable in untreated NAHAs. It is known that upon Aβ and/or IL-1β stimulation, astrocytes release IL-8 [4], a critical mediator for neutrophils-endothelial cells and astrocytes-endothelial cells interactions in CNS inflammatory reactions [5].

IL-1β is a relevant cofactor in AD development risk [6,7], AD neuroinflammation [8-10], and acts as a co-promoter of ICAM-1/sICAM-1 [11-16] and of RANTES [17,18] expression in NAHAs.

In experimental co-culture models, IL-3 released from astrocytes supports the growth of microglia cells, inducing them to remove Aβ fibrils, and exerting in such way a neuroprotective role [19].

IL-16 is a chemokine inducing migration across the BBB and proliferation of $CD4^{+}$ immune cells and promoting the expression of other proinflammatory cytokines including IL-1β and IL-6. In traumatic CNS injury astrocytes, microglia, and also neurons release this cytokine. IL-16 levels are found to be increased in AD patients as an expression of immune activation in response to the presence of Aβ deposits [20].

Moreover, the constitutive secretion of two other agents also involved in neuroinflammation, i.e. chemokine MCP-1 and metalloproteinase inhibitor TIMP-2, was unaffected in cultured Aβ ± NPS 2143-treated NAHAs [Table 1; Figure S1]. Hence, it is feasible that Aβ-treated microglia might be the main source of MCP-1 surpluses in AD brains [9]. TIMP-2 is amply expressed in adult CNS and its level is increased in CSF of AD patients, as regulator of metalloproteinases activity plays an anti-inflammatory and neuroprotective role [21].

In addition, our results show, rather unexpectedly, that 96-h after the experiments onset, a delayed stimulation of PDGF-BB secretion over both control and fAβ alone-exposed astrocytes took place in NAHAs co-treated with fAβ+NPS 2143 [Table 1, Supp Fig. 2]. PDGF-BB is thought to be an important angiogenic promoter in the tissue replacement phase after CNS injury and a neuroprotective agent, especially in cognitive dysfunction [22].

Clearly, more studies are needed to unravel the detailed mechanisms regulating the expression of these agents in NAHAs and to clarify their roles in human AD's neuroinflammation.

Other cytokines and chemokines arrays

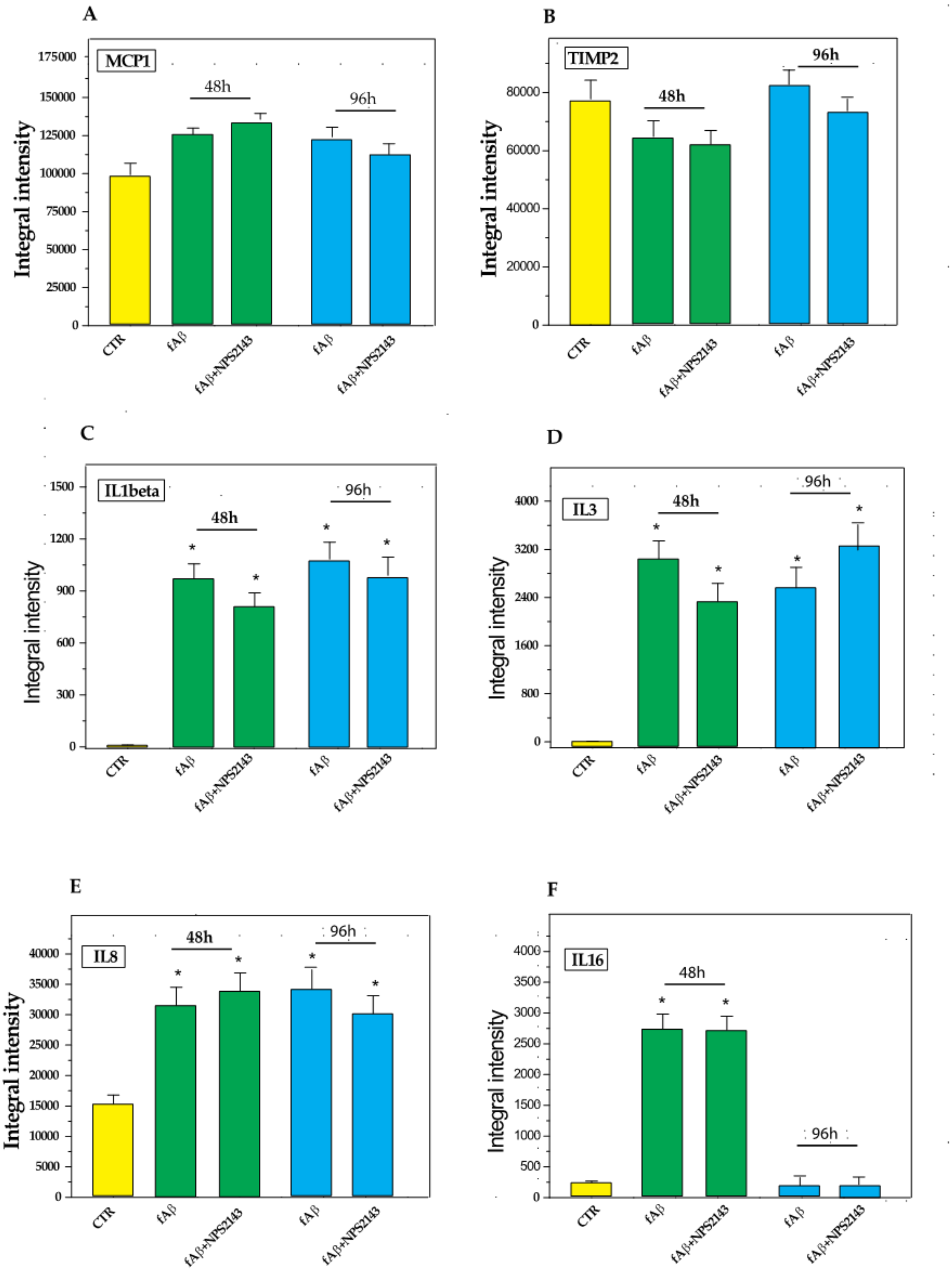

Figure S1.

Figure S1. Time-dependent expression of cytokines and chemokines (**A–F**) secreted by control and fAβ-treated NAHAs, the levels of which were unaffected by a co-treatment of fAβ with CaSR NAM NPS 2143. Each cytokine/chemokine was detected in the NAHA-conditioned media via membrane-based antibody array. The antibody array was analyzed with an Odissey™ (LI-COR) scanner and the positive staining intensities were quantified using the Image Studio™ (v.5.2) software. The integral intensities of the positive signals from each array were normalized via comparisons to corresponding positive controls. Results were expressed as mean values ± SEM. *P* values were calculated via one-way ANOVA followed by post hoc Tukey's test. * $P < 0.05$ *vs*. controls (CTR). fAβ, fAβ$_{25-35}$.

PDGF-BB Array

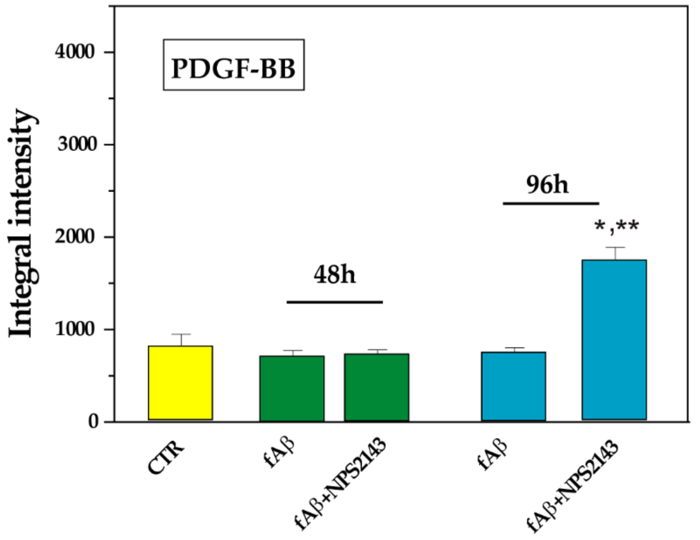

Figure S2

Figure S2. The secreted PDGF-BB levels pattern behaves differently from the all the other cytokines and chemokines we tested in the NAHA-conditioned media via membrane-based antibody array, because its basal levels did not change with respect to controls in fAβ-treated NAHAs for up to 96 h, but did increase at 96 h in fAβ+NPS 2143 co-treated NAHAs. The antibody array was analyzed with an Odissey™ (LI-COR) scanner and the positive staining intensities of PDGF-BB were quantified using the Image Studio™ (v.5.2) software. fAβ, fA$\beta_{25-35.}$ The integral intensities of the positive signals from each array were normalized via comparisons to corresponding positive controls. Results were expressed as mean values ± SEM. *P* values were calculated via one-way ANOVA followed by post hoc Tukey's test. * $P < 0.05$ vs. controls (CTR); ** $P < 0.05$ vs. fAβ. fAβ, fAβ_{25-35}.

S2. References:

1. Labzin, L.I.; Heneka, M.T.; Latz. E. Innate immunity and neurodegeneration. *Annu. Rev. Med*. **2018**, *69*, 437–449.
2. Becher, B.; Spath, S.; Goverman, J. Cytokine networks in neuroinflammation. *Nat. Rev. Immunol.* **2017**, *17*, 49–59.
3. Medeiros, R.; LaFerla, F.M. Astrocytes: conductors of the Alzheimer disease neuroinflammatory symphony. *Exp Neurol.* **2013**, *239*, 133–138.
4. Ashutosh; Kou, W.; Cotter, R.; Borgmann, K.; Wu, L.; Persidsky, R.; Sakhuja, N.; Ghorpade, A. CXCL8 protects human neurons from amyloid-beta-induced neurotoxicity: relevance to Alzheimer's disease. *Biochem. Biophys. Res. Commun.* **2011**, *412*, 565–571.
5. Liu, C.; Cui, G.; Zhu, M.; Kang, X.; Guo, H. Neuroinflammation in Alzheimer's disease: chemokines produced by astrocytes and chemokine receptors. *Int. J. Clin. Exp. Pathol*. **2014**, *7*, 8342–8355.
6. Croitoru-Lamoury, J.; Guillemin, G.J.; Boussin, F.D.; Mognetti, B.; Gigout, L.I.; Chéret, A.;Vaslin, B.; Le Grand, R.; Brew, B.J.; Dormont, D. Expression of chemokines and their receptors in human and simian astrocytes: evidence for a central role of TNF alpha and IFN gamma in CXCR4 and CCR5 modulation. *Glia*. **2003**, *41*, 354–370.
7. Chakrabarty, P.; Jansen-West, K.; Beccard, A.; Ceballos-Diaz, C.; Levites, Y.; Verbeeck, C.; Zubair, A.C.; Dickson, D.; Golde, T.E.; Das, P. Massive gliosis induced by interleukin-6 suppresses Abeta deposition in vivo: evidence against inflammation as a driving force for amyloid deposition. *FASEB J.* **2010**, *24*, 548–559.
8. Ambrosini, E.; Remoli, M.E.; Giacomini, E.; Rosicarelli, B.; Serafini, B.; Lande, R.; Aloisi, F.; Coccia, E.M. Astrocytes produce dendritic cell-attracting chemokines in vitro and in multiple sclerosis lesions. *J. Neuropathol. Exp. Neurol.* **2005**, *64*, 706–715.

9. Sokolova, A.; Hill, M.D.; Rahimi, F.; Warden, L.A.; Halliday, G.M.; Shepherd, C.E. Monocyte chemoattractant protein-1 plays a dominant role in the chronic inflammation observed in Alzheimer's disease. *Brain Pathol*. **2009**, *19*, 392–398.
10. Wang, W.Y.; Tan, M.S.; Yu, J.T.; Tan, L. Role of pro-inflammatory cytokines released from microglia in Alzheimer's disease. *Ann. Transl. Med.* **2015**, *3*, 136.
11. Lee, S.J.; Benveniste, E. N. Adhesion molecule expression and regulation on cells of the central nervous system. *J. Neuroimmunol.* **1999**, *98*, 77–88.
12. Schmal, H.; Czermak, B.J.; Lentsch, A.B., Bless, N.M.; Beck-Schimmer, B.; Friedl, H.P.; Ward, P.A. Soluble ICAM-1 activates lung macrophages and enhances lung injury. *J. Immunol.* **1998**, *161*, 3685–3693.
13. Ramos, T.N.; Bullard, D.C.; Barnum, S.R. ICAM-1: isoforms and phenotypes. *J. Immunol.* **2014**, *192*, 4469–4474.
14. Müller, N.The Role of Intercellular Adhesion Molecule-1 in the Pathogenesis of Psychiatric Disorders. *Front. Pharmacol.* **2019**, *10*, 1251.
15. Lawson, C.; Wolf, S. ICAM-1 signaling in endothelial cells. *Pharmacol. Rep*. **2009**, *61*, 22–32.
16. Wennstrom, M.; Nielsen, H. M.; Orhan, F.; Londos, E.; Minthon, L.; Erhardt, S. Kynurenic Acid levels in cerebrospinal fluid from patients with Alzheimer's disease or dementia with Lewy bodies. *Int. J. Tryptophan Res.* **2014**, *7*, 1–7.
17. Lin, M.S.; Hung, K.S.; Chiu, W.T.; Sun, Y.Y.; Tsai, S.H.; Lin, J.W.; Lee, Y.H. Curcumin enhances neuronal survival in N-methyl-d-aspartic acid toxicity by inducing RANTES expression in astrocytes via PI-3K and MAPK signaling pathways. *Prog. Neuropsychopharmacol. Biol. Psychiatry.* **2011**, *35,* 931–938.
18. Rivieccio, M.A.; John, G.R.; Song, X.; Suh, H.S.; Zhao, Y.; Lee, S.C.; Brosnan, C.F. The cytokine IL-1beta activates IFN response factor 3 in human fetal astrocytes in culture. *J. Immunol*. **2005**, *174*, 3719–3726.
19. Zambrano, A.; Otth, C.; Mujica, L.; Concha, I.I.; Maccioni, R.B. Interleukin-3 prevents neuronal death induced by amyloid peptide. *BMC Neurosci*. **2007**, *8*, 82.
20. Di Rosa, M.; Dell'Ombra, N.; Zambito, A.M.; Malaguarnera, M.; Nicoletti, F.; Malaguarnera, L. Chitotriosidase and inflammatory mediator levels in Alzheimer's disease and cerebrovascular dementia. *Eur. J. Neurosci*. **2006**, *23*, 2648–2656.
21. Lee, E.; Kim, H. The anti-inflammatory role of tissue inhibitor of metalloproteinase-2 in lipopolysaccharide-stimulated microglia. *J. Neuroinflammation* **2014**, *11*, 116.
22. Zhou, Z.; Wu, Q.; Lu, Y.; Zhang, X.; Lv, S.; Shao, J.; Zhou, Y.; Chen, J.; Hou, L.; Huang, C.; Zhang, X. Crosstalk between soluble PDGF-BB and PDGFR b promotes astrocytic activation and synaptic recovery in the hippocampus after subarachnoid hemorrhage. *FASEB J.* **2019**, *33*, 9588–9601.
